# Emerging Strategies for Immunotherapy of Solid Tumors Using Lipid‐Based Nanoparticles

**DOI:** 10.1002/advs.202305769

**Published:** 2023-12-06

**Authors:** Soraia Fernandes, Marco Cassani, Francesca Cavalieri, Giancarlo Forte, Frank Caruso

**Affiliations:** ^1^ Center for Translational Medicine (CTM) International Clinical Research Centre (ICRC) St. Anne Hospital Brno 656 91 Czech Republic; ^2^ Department of Chemical Engineering The University of Melbourne Parkville Victoria 3010 Australia; ^3^ School of Science RMIT University Melbourne Victoria 3000 Australia; ^4^ Dipartimento di Scienze e Tecnologie Chimiche Universita di Roma “Tor Vergata” Via della Ricerca Scientifica 1 Rome 00133 Italy; ^5^ School of Cardiovascular and Metabolic Medicine & Sciences King's College London London SE5 9NU UK

**Keywords:** ECM, immunotherapy, lipid‐based nanoparticles, mechanotherapy, tumor

## Abstract

The application of lipid‐based nanoparticles for COVID‐19 vaccines and transthyretin‐mediated amyloidosis treatment have highlighted their potential for translation to cancer therapy. However, their use in delivering drugs to solid tumors is limited by ineffective targeting, heterogeneous organ distribution, systemic inflammatory responses, and insufficient drug accumulation at the tumor. Instead, the use of lipid‐based nanoparticles to remotely activate immune system responses is an emerging effective strategy. Despite this approach showing potential for treating hematological cancers, its application to treat solid tumors is hampered by the selection of eligible targets, tumor heterogeneity, and ineffective penetration of activated T cells within the tumor. Notwithstanding, the use of lipid‐based nanoparticles for immunotherapy is projected to revolutionize cancer therapy, with the ultimate goal of rendering cancer a chronic disease. However, the translational success is likely to depend on the use of predictive tumor models in preclinical studies, simulating the complexity of the tumor microenvironment (e.g., the fibrotic extracellular matrix that impairs therapeutic outcomes) and stimulating tumor progression. This review compiles recent advances in the field of antitumor lipid‐based nanoparticles and highlights emerging therapeutic approaches (e.g., mechanotherapy) to modulate tumor stiffness and improve T cell infiltration, and the use of organoids to better guide therapeutic outcomes.

## Graphical abstract

The implementation of advanced preclinical models such as tumor organoids, incorporating immune system cells and tumor extracellular matrix, will contribute to the development of innovative antitumor therapies such as mechanotherapy‐assisted lipid‐based immunotherapy.

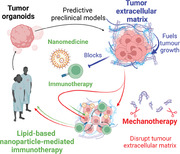

## 1 Lipid‐Based Nanoparticles in Nanomedicine

**Figure 1:**
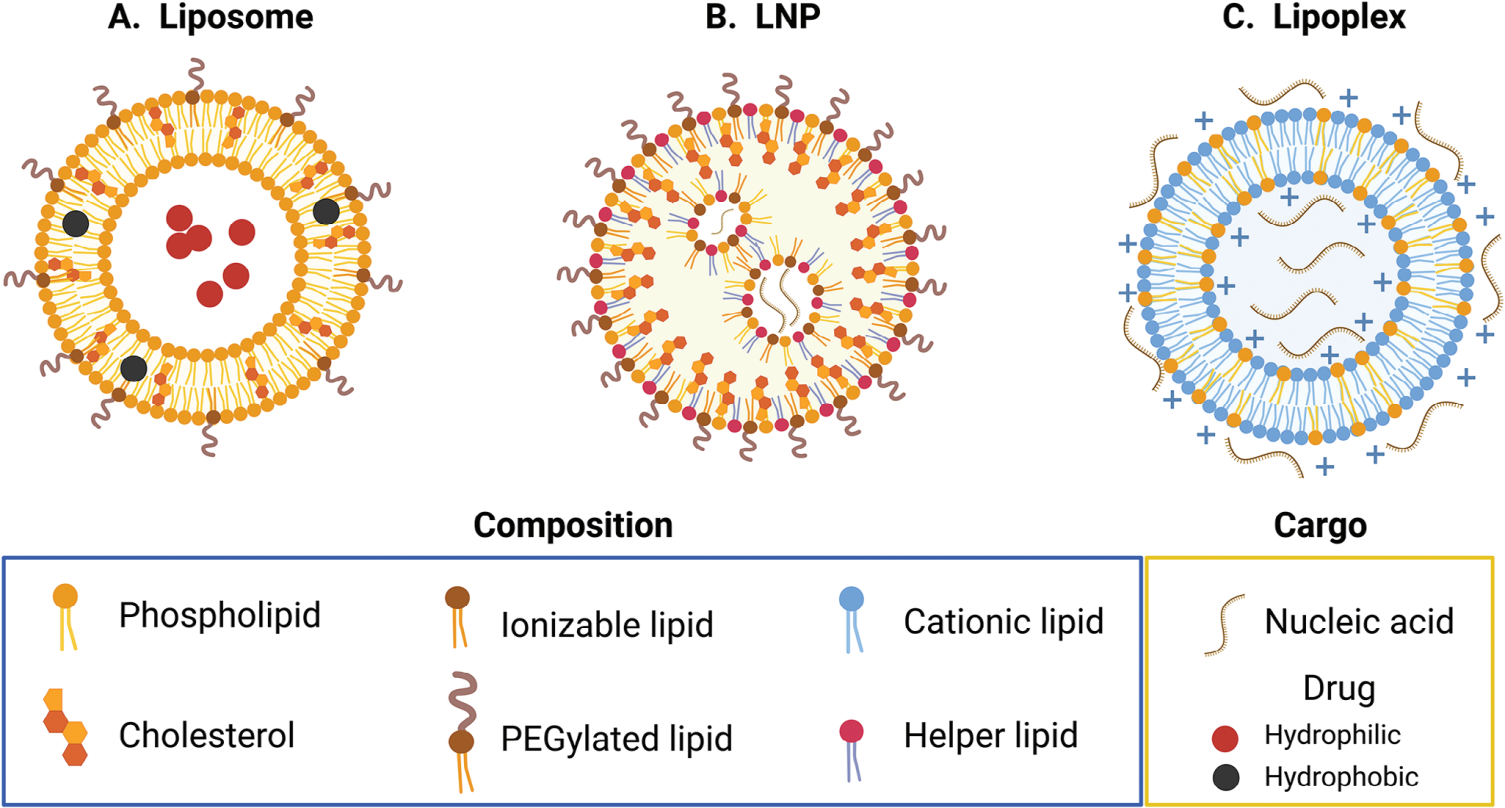
Schematic representation of different lipid‐based nanoparticles. A) Liposomes are made of one or more lipid bilayers composed of phospholipids (e.g., phosphatidylcholines and phosphatidylethanolamines) and stabilizing constituents (i.e., cholesterol). Conventionally, liposomes have been used to encapsulate hydrophobic chemotherapeutic drugs (e.g., Doxil). B) LNPs are composed of cholesterol and a mixture of lipids, including functional ionizable, helper, and PEGylated lipids. Their current main application is the delivery of nucleic acids. Alternatively, C) lipoplex‐formulated nanoparticles can also be used for the delivery of nucleic acids by mixing DNA/RNA and ionizable/cationic lipids. We note that other classes of lipid‐based nanoparticles have been synthesized, such as cubosomes, solid–lipid nanoparticles, and nanostructured lipid carriers. However, in this figure, we provide an overview of the three main categories that have been used in nanomedicine and immunotherapy applications and that are discussed in this review. Created with Biorender.com.

Since their discovery in the 1960s, lipid‐based nanoparticles (**Figure** **1**) have attracted interest owing to their ability to encapsulate and deliver therapeutic molecules, i.e., hydrophobic small molecule drugs^[^ ^1^ ^]^ and hydrophilic biomacromolecules, such as RNA,^[^ ^2^ ^]^ DNA,^[^ ^2^, ^3^ ^]^ and proteins,^[^ ^4^ ^]^ to target specific cellular populations. Liposomes (Figure 1A), the first class of lipid‐based nanoparticles employed in nanomedicine, are spherical vesicles made of one or more lipid bilayers composed of phospholipids, such as phosphatidylcholines and phosphatidylethanolamines, and stabilizing constituents, such as cholesterol.^[^ ^5^ ^]^ Over the years, the composition and physicochemical properties of lipid‐based nanoparticles have been improved to overcome issues mainly related to their inadequate stability in complex biological fluids, which ultimately lead to the premature release of the encapsulated cargo and the occurrence of adverse side effects.^[^ ^5^ ^]^ These advancements have led to the development of the so‐called lipid nanoparticles (LNPs, Figure 1B) synthesized from a mixture of functional lipids that can include cationic or ionizable lipids, helper lipids, poly(ethylene glycol)‐modified lipids (PEGylated lipids), and cholesterol.^[^ ^6^ ^]^ Cholesterol imparts structural rigidity to LNPs, the cationic or ionizable lipids enable the encapsulation and endosomal escape of the nucleic acid payload, whereas the helper lipids improve LNP stability and fusogenicity.^[^ ^6^, ^7^ ^]^ By preventing nonspecific serum protein adsorption and nanoparticle aggregation, PEGylated lipids improve the pharmacokinetics of LNPs.^[^ ^8^ ^]^ The specific composition and morphology of LNPs can vary depending on the intended application and the type of payload being delivered. For instance, the structure of LNPs containing small‐interfering RNA (siRNA) or messenger RNA (mRNA) is characterized by a hydrophobic electron dense core, consisting of inverted micelles of lipid encapsulating hydrated nucleic acids, surrounded by a coating of PEGylated lipids.^[^ ^9^ ^]^ Recently, the role of lipid composition in conferring specific physicochemical features to the nanoformulation, as well as in determining its organ biodistribution after intravenous (IV) administration has emerged as a key feature to direct the action of lipid‐based therapies to specific tissues.^[^ ^10^ ^]^ Cationic lipids can also be mixed with RNA and DNA to form lipoplexes (Figure 1C).^[^ ^11^ ^]^ Although the first generation of cationic lipids has proven useful for in vitro transfection purposes, the use of such cationic lipids in vivo is limited owing to their large size (>1 µm diameter), instability, positive surface charge, and dose‐limiting toxic side effects.^[^ ^12^ ^]^ However, a new generation of lipoplex–mRNA constructs are currently under evaluation in clinical trials for the treatment of advanced solid tumors.^[^ ^13^ ^]^

LNPs have shown higher flexibility in terms of cargo loading (small drug molecules and large nucleic acids), encapsulation efficiency (90–100%), and scalability when compared to other nanoparticles based on polymeric or inorganic materials.^[^ ^14^ ^]^ Therefore, LNPs are currently a highly attractive platform for developing novel therapeutic strategies to treat cancer,^[^ ^2^, ^15^ ^]^ nervous system disorders,^[^ ^16^ ^]^ and infectious diseases.^[^ ^9b^ ^]^

## 2 Direct Targeting of Solid Tumors with Lipid‐Based Nanoparticles

Chemotherapeutic drugs are among the first molecules to be proposed as payloads for lipid‐based nanoparticles, by virtue of their availability and well‐known mechanism of action.^[^ ^17^ ^]^ As many of these drugs are hydrophobic or sparingly soluble in physiological media, they are well‐suited for incorporation into lipid‐based delivery systems to enhance their biodistribution and bioavailability, and therefore boost their therapeutic efficacy while reducing off‐target accumulation and dosing regimen.^[^ ^18^ ^]^ For these reasons, over the past three decades and since the approval of the first nanodrug, Doxil, in 1995,^[^ ^19^ ^]^ the clinical applications of lipid‐based nanoparticles have surged. Two of the most recently approved lipid‐based nanodrugs, Onivyde and Vyxeos (or CPX‐351), demonstrate the concept of drug encapsulation into lipid‐based nanoparticles, aiming at increasing the efficacy of chemotherapy drugs while reducing the toxicity associated with traditional formulations.^[^ ^20^ ^]^ Onivyde is a liposomal formulation of irinotecan, approved by the US Food and Drug Administration (FDA) in 2015 for the treatment of metastatic pancreatic cancer in combination with leucorvin and fluorouracil as a chemotherapeutic regimen.^[^ ^21^ ^]^ In addition to their use as a stand‐alone therapy or in combination with standard chemotherapy, lipid‐based nanoparticles allow the loading of different chemotherapeutic agents within the same carrier, thereby potentially resulting in more effective treatments. In this regard, Vyxeos, a liposomal formulation designed for the delivery of cytarabine and daunorubicin, was developed for the treatment of acute myeloid leukemia and approved by the FDA in 2017.^[^ ^22^ ^]^ We note that different approaches involving lipid‐based nanoparticles that are responsive to external physical stimuli can be used for delivering therapeutics to solid tumors. For example, stimuli‐responsive lipid‐based nanosystems, such as lipid‐based magnetic nanovectors that are responsive to an external magnetic field and carry chemotherapeutic drugs, have been studied at the preclinical stage. The synergistic therapy provided by these nanovectors, i.e., hyperthermia combined with in situ drug delivery after local injection at the tumor site, has revealed prolonged survival of animals suffering from glioblastoma multiforme.^[^ ^23^ ^]^ A detailed description of these nanosystems is outside the scope of the present review but detailed information on these therapeutic strategies is provided elsewhere.^[^ ^24^ ^]^

Despite the inherent benefits of using nanocarriers for the delivery of chemotherapy drugs, many of the existing nanodrug formulations do not demonstrate an improvement in patient overall survival when compared to conventional free drug regimens. In addition, their benefits, although not negligible, fail to meet the initial expectations and are limited, in most cases, to reducing off‐target side effects.^[^ ^25^ ^]^ It has been extensively reported that only a small fraction (<0.6%) of the administered nanoparticles effectively accumulates into solid tumors, with modest improvements and uncertain outcomes even when targeting moieties are used.^[^ ^26^ ^]^

Lipid‐based nanoparticles have also been used for the delivery of therapeutic nucleic acids, i.e., siRNA and microRNA (miRNA), antisense oligonucleotides, and mRNA, with anticancer activity. The concept of mRNA delivery was introduced more than three decades ago, aiming at modulating the expression of proteins of interest in the target T cells to reestablish their physiological function.^[^ ^27^ ^]^ Subsequently, the discovery of RNA interference mechanisms opened the possibility of exploiting siRNA molecules to silence specific disease‐related genes.^[^ ^28^ ^]^

The direct administration of naked nucleic acids is hampered by: i) their possible degradation by nucleases; ii) their inability to cross biological membranes; and iii) possible undesired immunological reactions to nucleic acids.^[^ ^29^ ^]^ Hence, the incorporation and protection of therapeutic nucleic acids into lipid‐based delivery systems, including liposomes and LNPs, have been widely explored in the past two decades. The significant progress made in designing and engineering lipid‐based drug delivery systems with optimal chemical properties and composition, as well as increased stability and efficacy has enabled the clinical translation of LNP‐mediated gene therapy.

Two LNP formulations for siRNA delivery, Patisiran (Onpattro) and Givosiran (Givlaari), have been approved for the treatment of transthyretin‐mediated amyloidosis (in 2018) and acute hepatic porphyria (in 2019), respectively.^[^ ^16^, ^30^ ^]^ More recently, the developments achieved in the field of nanoparticles have resulted in several LNP formulations being evaluated in clinical trials for solid tumor gene therapy including the delivery of antisense nucleic acids (WGI‐0301), plasmid DNA (Reqorsa), siRNA (TKM‐080301), miRNA (INT‐1B3), and mRNA (OTX‐2002), as listed in **Table** **1**. Generally, these formulations are administered systemically by IV injection and are expected to act directly upon the tumor cells or the tumor microenvironment (TME), inhibiting tumor growth by downregulating the expression of oncogenes or upregulating the expression of tumor suppressor genes. During Phase 1 and Phase 2 clinical trials, the safety and efficacy of the nanotherapeutics are evaluated. For instance, clinical trials conducted on the administration of TKM‐080301 have revealed that most of these LNPs accumulate in the liver (66–83%). The accumulation at the tumor site is expected to occur by the enhanced permeability and retention (EPR) effect. However, the EPR effect has been challenged lately by evidence showing that only 0.6% of nanoparticles is retained at the tumor site.^[^ ^26a^ ^]^ Thus, although TKM‐080301 is well tolerated, its antitumor effect is limited.^[^ ^31^ ^]^ Overall, the limited tumor extravasation and nonspecific biodistribution still present major translational challenges for specific solid tumor treatment.

**Table 1:** Selection of clinical trials of LNPs for treating solid tumors.

Identifier^a^ ^)^	Condition	Composition	Mode of action	A.R.	Status
NCT05267899	Advanced solid tumors	**LNP–antisense ODN** (WGI‐0301)	20‐mer antisense oligonucleotide against proto‐oncogene Akt‐1 (Archexin) inhibiting PI3K/Akt‐mediated signaling	N.A.	Phase 1 (recruiting)
NCT01437007	Primary or secondary liver cancer	**LNP–siRNA** (TKM‐080301)	siRNA against proto‐oncogene PLK1 overexpressed in HCC	IV	Phase 1 (completed)
NCT02191878	Advanced liver cancer				Phase 1 Phase 2 (completed)
NCT05062980	NSCLC	**LNP–plasmid** (quaratusugene ozeplasmid – Reqorsa)	DNA plasmid with the TUSC2 (FUS‐1) tumor suppressor gene. + pembrolizumab (anti‐PD‐1)	IV	Phase 1 Phase 2 (recruiting)
NCT04486833	Advanced lung cancer		+ EGFR‐targeted drug osimertinib or platinum‐based chemotherapy		Phase 1 Phase 2 (recruiting)
NCT05703971	Extensive stage – small cell lung cancer		+ Atezolizumab (anti‐PD‐L1)		Phase 1 Phase 2 (recruiting)
NCT04675996	Advanced solid tumors	**LNP–miRNA** (INT‐1B3)	miR‐193a‐3p with multitarget mechanism (antiproliferative, antimetastatic, antimigration, cell cycle disruption, proapoptotic, TME modulation) leading to significant induction of T cell‐mediated immune response	IV	Phase 1 (recruiting)
NCT05497453	HCC and solid tumors associated with MYC oncogene	**LNP–mRNA** (OTX‐2002)	Biscintronic mRNA encoding for epigenomic controller proteins ZF‐DNMT and ZF‐KRAB that inhibit the expression of proto‐oncogenes. Monotherapy or combination with TKIs or checkpoint inhibitors	IV	Phase 1 Phase 2 (recruiting)

^a)^ Data obtained from reference.^[^ ^36^ ^]^ AR, Administration route; LNP, lipid nanoparticle; ODN, oligodeoxynucleotide; Akt‐1, AKT serine/threonine kinase 1; PI3K, phosphatidylinositol 3‐kinase; N.A., not applicable; PLK1, polo‐like kinase 1; HCC, hepatocellular carcinoma; IV, intravenous administration; NSCLC, nonsmall cell lung cancer; TUSC2 (FUS‐1), tumor suppressor 2, mitochondrial calcium regulator; PD‐1, programmed cell death protein 1; EGFR, epidermal growth factor receptor; PD‐L1, programmed death ligand; TME, tumor microenvironment; MYC, proto‐oncogene, transcription factor; ZF‐DNMT, zinc finger‐DNA‐methyltransferase; ZF‐KRAB, zinc‐finger‐Krüppel‐associated box domain; TKI, tyrosine kinase inhibitor.

Rather than inhibiting the expression of pathological gene variants, mRNA therapy has been developed with the aim to (i) promote the expression of missing or downregulated proteins or (ii) induce the expression of specific antigens that can initiate an immune response.^[^ ^32^ ^]^ Intuitively, while some diseases, such as cystic fibrosis^[^ ^33^ ^]^ and phenylketonuria,^[^ ^34^ ^]^ can only be treated with the reestablishment of cell functionality, infection diseases and cancers can greatly benefit from immunotherapy. Accordingly, the first‐approved vaccines against COVID‐19, Pfizer‐BioNTech BNT162b2 and Moderna mRNA‐1273, use LNPs to deliver mRNA encoding the spike protein of the SARS‐CoV‐2 virus, which stimulates an immune response in the body, providing ∼90% protection against infection.^[^ ^35^ ^]^

## 3 LNPs for Immunotherapy

The success of mRNA vaccines and the short time elapsed from their design to their commercialization, accompanied by the emergency use authorization granted by the FDA, has sparked great optimism for the use of LNPs to treat several other disorders in the near future.^[^ ^37^ ^]^ Along with the research efforts applied to develop mRNA vaccines, several immunotherapies such as immune checkpoint inhibitors (ICIs), bispecific T cell engagers (BiTEs), or chimeric antigen receptor (CAR) technology have reached the clinic in the last few years, improving cancer treatment.^[^ ^38^ ^]^

Notwithstanding the success of immunotherapies in recent years, several challenges have emerged, such as interpatient heterogeneous responses, selection of suitable therapeutic targets, and safety concerns.^[^ ^39^ ^]^ For example, ICIs have demonstrated efficacy in fewer than half of the patients deemed suitable for the treatment (which represents half of the patients suffering from cancer), BiTEs have short life in the body, and chimeric antigen receptor‐T (CAR‐T) cell therapy is limited by the availability of appropriate targets and their efficiency against solid tumors is limited.^[^ ^40^ ^]^ In addition, the high production costs and the requirement of specialized clinics and facilities limit the application of immunotherapy. These limitations have contributed to research focused on nanovaccines and mRNA delivery systems that can trigger immune responses potentially against any type of cancer. Thus, the development of LNPs as cancer nanovaccines has offered novel solutions for treating, or even prevent, cancer by overcoming limited response issues and safety‐related concerns, owing to the versatility of LNP vector design and mRNA synthesis.^[^ ^41^ ^]^ Several LNP and lipoplex formulations for the delivery of mRNA encoding patient‐specific neoantigens are currently undergoing clinical trials, aiming at personalizing therapeutic interventions as stand‐alone treatments or more efficiently in combination with chemotherapies, antibody‐dependent cell‐mediated cytotoxicity, ICIs, or other immunotherapies (**Table** **2**). During Phase 1 of the clinical trials, safety and tolerability of the nanoparticles are evaluated to ensure the absence of severe side effects. During Phase 2, drug efficacy is studied. Table 2 presents several liposome, lipoplexe, and LNP formulations for the delivery of mRNA against specific inflammatory interleukins (ILs), such as IL‐12 (NCT03946800), mRNA vaccines against several tumor‐associated antigens (TAAs) (e.g., NCT04503278, NCT03948763, and NCT03480152), or other proteins known to stimulate immune responses, e.g., OX40L (NCT03323398).

**Table 2:** Selection of clinical trials of lipid‐based NPs for cancer immunotherapy.

Identifier^a^ ^)^	Condition	Composition	Mode of action	A.R.	Status
NCT04486378	Pancreatic cancer	**Lipoplex–mRNA** (Autogene cevumeran ‐ RO7198457)	mRNA‐based and patient‐specific neoadjuvant with immunostimulatory and antineoplastic activities	IV	Phase 2
NCT03289962	Advanced and metastatic tumors		+ Atezolizumab		Phase 1
NCT04161755	Advanced melanoma		+ Atezolizumab + FOLFIRINOX		Phase 1
NCT03815058	Colorectal cancer		+ Pembrolizumab		Phase 2
NCT04503278	Advanced CLDN6‐positive tumors	**Lipoplex–mRNA** (BNT211)	mRNA vaccine encoding CLDN6 TAA for the stimulation of CAR‐T in vivo. In combination with CAR‐T therapy (CARVac)	IV	Phase 1 Phase 2 (recruiting)
NCT04683939	Unresectable or metastatic CLDN18.2‐positive gastric, pancreatic, ovarian, and biliary tract tumors	**Lipoplex–mRNA** (BNT141)	mRNA encoding mAb against CLDN18.2 (RiboMabs)	IV	Phase 1 Phase 2
NCT03948763	NSCLC, colorectal cancer, pancreatic adenocarcinoma	**LNP–mRNA vaccine** (mRNA‐5671 – V941)	mRNA vaccine encoding four TAAs of the most common KRAS mutations in cancer. Monotherapy or in combination with pembrolizumab	IM	Phase 1 (completed)
NCT03323398	Relapsed solid tumors, lymphoma, and ovarian cancer	**LNP–mRNA** (mRNA‐2416)	mRNA encoding human OX40L. Monotherapy or in combination with durvalumab (anti‐PD‐L1)	IT	Phase 1 Phase 2 (terminated)
NCT03739931	Relapsed solid tumors or lymphoma	**LNP–mRNA** (mRNA‐2752)	mRNA encoding human OX40L, IL‐23 and IL‐36γ. Monotherapy or in combination with durvalumab	IT	Phase 1
NCT03946800	Advanced solid tumors	**LNP–mRNA** (MEDI1191)	mRNA encoding IL‐12. Monotherapy or in combination with durvalumab	IT	Phase 1
NCT04455620	Solid tumors	**LNP–mRNA** (BNT151)	mRNA encoding IL‐2 (RiboCytokines)	IV	Phase 1 Phase 2
NCT04710043		**LNP–mRNA** (BNT152 + BNT153)	mRNA encoding a combination of IL‐2 and IL‐7 (RiboCytokines)		Phase 1
NCT03480152	Melanoma, colon, gastrointestinal, genitourinary cancer, and HCC	**Liposome–mRNA vaccine** (mRNA‐4650)	Personalized mRNA vaccine encoding up to 20 TAAs in combination with pembrolizumab	IM	Phase 1 Phase 2 (terminated)
NCT03897881	Melanoma	**Liposome–mRNA vaccine** (mRNA‐4157/V940)	Personalized mRNA vaccine encoding up to 34 TAAs in combination with pembrolizumab	IV	Phase 2 (recruiting)
NCT04526899 (Lipo‐MERIT)	Melanoma	**Liposome–mRNA vaccine** (BNT111)	mRNA vaccine encoding four nonmutant TAAs (MAGE‐A3, NY‐ESO‐1, tyrosinase, and TPTE). Monotherapy or in combination with Cemiplimab (anti‐PD‐1)	IV	Phase 2
NCT04382898 (PRO‐MERIT)	Prostate cancer	**Liposome‐mRNA vaccine** (BNT112)	mRNA vaccine encoding a set of five prostate TAAs antigens (PAP, PSA, and three undisclosed antigens). Monotherapy or in combination with Cemiplimab	IV	Phase 1 Phase 2
NCT04534205	HPV‐positive head and neck squamous cell carcinoma	**Liposome–mRNA vaccine** (BNT113)	mRNA vaccine encoding for HPV‐16 oncoproteins E6 and E7 in combination with pembrolizumab	IV	Phase 2
NCT02316457 (TNBC MERIT)	TNBC	**Liposome–RNA vaccine** (BNT114)	Personalized mRNA vaccine encoding up to 20 TAAs	IV	Phase 1
NCT04163094 (OLIVIA)	Ovarian cancer	**Liposome–mRNA vaccine** (BNT115)	mRNA vaccine encoding three TAAs in combination with neo‐adjuvant chemotherapy including cycles of carboplatin/paclitaxel and interval surgery	IV	Phase 1
NCT05142189 (LuCa‐MERIT‐1)	NSCLC	**Liposome–mRNA vaccine** (BNT116)	mRNA vaccine encoding a set of six TAAs (undisclosed). Monotherapy or in combination with Cemiplimab or docetaxel	IV	Phase 1

^a)^ Data obtained from reference.^[^ ^36^ ^]^ A.R., Administration route; IV, intravenous administration; FOLFIRINOX, therapeutic regimen of leucovorin calcium (folinic acid), fluorouracil, irinotecan hydrochloride, and oxaliplatin; CLDN6, claudin 6; TAA, tumor‐associated antigen; CAR‐T, chimeric antigen receptor‐T; mAb, monoclonal antibody; CLDN18.2, claudin 18.2; NSCLC, nonsmall cell lung cancer; LNP, lipid nanoparticle; KRAS, proto‐oncogene, small GTPase part of the RAS/MAPK pathway; IM, intramuscular administration; OX40L, a membrane‐bound member of the tumor necrosis factor superfamily, known to costimulate immune response; PD‐L1, programmed death ligand; IT, intratumoral administration; IL, interleukin; HCC, hepatocellular carcinoma; MERIT, mutanome‐engineered RNA immunotherapy; MAGE‐A3, melanoma‐associated antigen 3; NY‐ESO‐1, New York esophageal squamous cell carcinoma 1; TPTE, transmembrane phosphatase with tensin homology; PD‐1, programmed cell death protein 1; PAP, prostatic acid phosphatase; PSA, prostatic‐specific antigen; HPV, human papilloma virus; TNBC, triple negative breast cancer.

**Figure 2:**
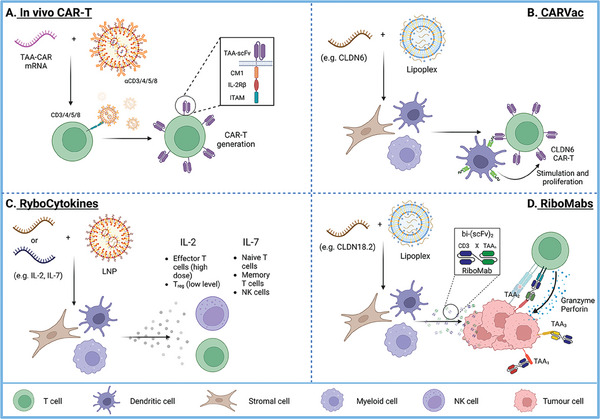
Advanced immunotherapeutic approaches. A) In vivo CAR‐T involves LNPs that target T cells (using specific antibodies, e.g., CD3, CD4, CD6, and CD8) for the delivery of mRNAs against TAAs that can produce CAR‐T cells in vivo. B) CARVac technology entails lipoplex formulations carrying mRNA encoding for tumor‐specific antigens (e.g., CLDN6) to stimulate the proliferation and activation of CAR‐T cells engineered against the same antigen. C) RiboCytokine formulations contain cytokine‐encoding mRNA to induce the secretion of immunostimulating cytokines (e.g., IL‐2 and IL7) and enhance the antitumor activity of T and NK immune cells. Different levels of cytokines may stimulate the immune system differently (e.g., high doses of IL‐2 can effectively activate effector T cells, whereas sustained low doses can promote their differentiation into T_reg_, with opposite effects).^[^ ^46^ ^]^ D) RiboMab technology uses mRNA encoding bispecific antibodies that function as T cell‐engager antibodies. The latter recruit cytotoxic T cells to tumor cells to induce target‐dependent T cell activation and tumor cell lysis through the local release of granzyme and perforin proteases. Perforin forms transmembrane pores for the subsequent diffusion of granzymes to the target cell cytosol, initiating cellular apoptosis. TAA, tumor‐associated antigen; CM1, costimulatory molecule 1; IL, interleukin; IL‐2Rβ, IL2 receptor β; ITAM, immunoreceptor tyrosine‐based activation motif; CLDN6, claudin 6; LNP, lipid nanoparticle; T_reg_, regulatory T cells; NK, natural killer; CLDN18.2, claudin 18.2; Created with BioRender.com.

Recently, the application of in vivo CAR‐T (**Figure** **2**A), not only limited to cancer treatment, has emerged as a promising approach to reduce the cost of CAR‐T therapy and potentially its side effects, thus extending its applicability and accessibility.^[^ ^42^ ^]^ The use of LNPs to deliver in vivo mRNA encoding CAR has been successfully demonstrated at the preclinical stage by targeting T cells with different antibody‐functionalized LNPs against CD3/4/5/8 receptors.^[^ ^43^ ^]^ To overcome CAR‐T therapy limitations, such as inefficient responses, persistence and survival of engineered T cells after administration, a costimulatory mRNA vaccine system (CARVac) has been developed to boost CAR‐T cell activity (Figure 2B).^[^ ^44^ ^]^ In this case, the lipoplex mRNA vaccine promotes the expression of claudin 6 (CLDN6) on the surface of dendritic cells (DCs), which in turn enhance the efficacy of CLDN6–CAR‐T cells tumor therapy.^[^ ^44^ ^]^ Additionally, a new class of immunotherapeutics known as RiboCytokines (Figure 2C, see NCT04455620 and NCT04710043 in Table 2) and RiboMabs (Figure 2D, see NCT04683939 in Table 2) use bispecific antibodies or cytokine‐encoding mRNA, respectively, to stimulate their production in patients and elicit an immune response against tumor cells.^[^ ^13^, ^45^ ^]^ This new generation of immunotherapies contains naturally occurring building blocks that confer stability and lower the risk of unwanted immune reactions, which represent two of the main challenges in the development of safe and effective mRNA therapeutics. Their use for the treatment of unresectable solid tumors or metastasis is currently under clinical trial (see Table 2).

Nonetheless, there remain several challenges related to the transient nature of mRNA and inherent to the nature of the TME in solid cancers, including its immunosuppressive features. Once activated against the desired antigen, T cells and other effector immune cells are subjected to a highly immunosuppressive environment at the tumor site, which not only inhibits immune cells through chemokines, cytokines, and metabolic by‐products but also sustains the production and deposition of a dense extracellular matrix (ECM) that is able to hamper the diffusion of the effector immune cells throughout the tumor tissue.^[^ ^47^ ^]^

## 4 Translational Challenges

### 4.1 Biological Barriers to Targeting Solid Tumors

Solid tumors are dynamic tissues composed of cancer cells, ECM, and stromal cells including endothelial cells, cancer‐associated fibroblasts (CAFs), and immune cells, which may be resident or infiltrating.^[^ ^48^ ^]^ The complex orchestration among all these components forms the so‐called TME with characteristic hallmarks common among most solid tumors.^[^ ^49^ ^]^ The TME is constantly evolving, promoting tumor progression by ensuring cancer cell survival and eventually migration/invasion of metastatic sites.^[^ ^50^ ^]^ Stromal cells are recruited by cancer cells from neighboring tissues to secrete soluble factors responsible for promoting angiogenesis, cancer cell growth, and remodel of the ECM.^[^ ^50^ ^]^ The ECM is constantly remodeled, with CAFs being its major effector.^[^ ^49^, ^51^ ^]^ CAFs differ from nonpathological fibroblasts owing to their enhanced activity, which is translated into excessive ECM protein deposition and remodeling. This in turn results in desmoplasia, a condition in which the ECM acquires a fibrotic‐like phenotype, which undergoes continuous remodeling and degradation to facilitate cancer migration.^[^ ^51^, ^52^ ^]^

Noteworthy, the ECM is the major constituent of solid tumors, representing 60% of the entire tumor mass with ∼300 proteins composing the matrisome.^[^ ^48^, ^49^ ^]^ Two different parts of the ECM can be distinguished: (i) the interstitial ECM, a three‐dimensional (3D) network interconnecting cells in the stroma, and (ii) the basement membrane, which keeps the cells connected to the ECM.^[^ ^49^ ^]^ The basement membrane is composed mainly of collagen IV and laminin and is essential for maintaining epithelial polarity and organization of healthy tissues. However, tumorigenesis leads to a loss of basement membrane organization, while promoting the expression of major ECM components involved in cancer progression such as collagen I, fibronectin, and matrix metalloproteinases (MMPs).^[^ ^53^ ^]^ MMPs play a key role in ECM degradation and, therefore, contribute to enhancing metastasis dissemination.^[^ ^49^, ^53^ ^]^ In addition to the physical support provided to cancer cells, the ECM serves as a reservoir for secreted factors including cytokines and growth factors, e.g., pro‐angiogenic factors.^[^ ^49^ ^]^ These biophysical and biochemical changes in the TME affect cell signaling by promoting cancer cell epithelial‐to‐mesenchymal transition (EMT), thus establishing a positive loop between TME and cancer cells that promotes solid tumor progression.^[^ ^54^ ^]^ These pathological alterations and in particular the existence of an abundant ECM represent a barrier to realizing the homogeneous and effective penetration of nanoparticles and molecules (such as antibodies, peptides, and nucleic acids) into the tumor core.^[^ ^55^ ^]^ In addition, the ECM hampers immune cell infiltration, hence preventing immune patrolling and tumor clearance, ultimately facilitating immune escape and tumor progression.^[^ ^56^ ^]^ Therefore, this issue needs to be addressed before developing immunotherapies in which targeted activated T cells would face infiltration issues within the fibrotic TME. The TME contributes to tumor immune system evasion by different mechanisms: (i) blocking DC activation; (ii) deregulating immune cells recruitment; (3) sequestering T cells at the ECM; (4) and inducing T cell exhaustion.^[^ ^57^ ^]^ Moreover, the excessive release of pro‐angiogenic factors, e.g., vascular endothelial growth factor (VEGF)‐A, often results in the formation of undeveloped vasculature in fast‐growing tumors.^[^ ^58^ ^]^ This distorted organization of irregular tortuous new blood vessels can also limit T cell extravasation at the tumor site, due to uneven flow, as well as disrupt endothelial junctions and cytoskeleton alterations of the endothelial cells, which ultimately prevents their transmigration.^[^ ^58^, ^59^ ^]^

**Figure 3:**
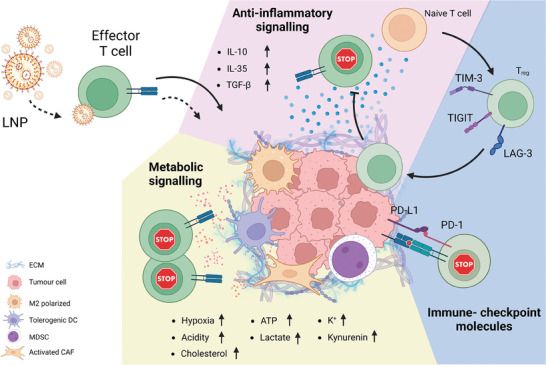
Schematic representation of immunosuppressive activity of TME. The TME suppresses effector T cell activity by (i) releasing anti‐inflammatory signals, i.e., cytokines and growth factors, (ii) modulating the metabolic signaling (e.g., inducing hypoxia), and (iii) regulating immune checkpoint molecule expression, e.g., PD‐L1. LNP, lipid nanoparticle; ECM, extracellular matrix; DC, dendritic cell; MDSC, myeloid‐derived suppressive cell; CAF, cancer‐associated fibroblast; T_reg_, regulatory T cells; PD‐L1, programmed death ligand 1; PD‐1, programmed cell death protein 1; ATP, adenosine triphosphate. Created with BioRender.com.

During tumor growth, several molecules and cytokines are cooperatively produced by the different types of cells residing at the TME, i.e., the cancer cells, CAFs, endothelial cells, and immune cells (**Figure** **3**). In particular, the tumor immune microenvironment (TIME) is enriched with many different immune cells including myeloid‐derived suppressive cells (MDSCs) (macrophages and monocytes) and lymphocytes (regulatory T cells (T_reg_) and naïve T cells) (Figure 3).^[^ ^60^ ^]^ T_reg_ cells, responsible for regulating or suppressing the immune system activity, and MDSCs are recruited to the tumor site in response to the oncogenic‐driven secretion of several factors and cytokines. In the TIME, the continuous release of onco‐cytokines dictates their immunosuppressive function, contributing to the inhibition of antitumor immunity.^[^ ^60^ ^]^ For instance, pro‐tumorigenic M2‐polarized macrophages and tolerogenic DCs promote TME remodeling, in a process that leads to the confinement of effector T cells in the stroma and hampers their infiltration into the tumor core and thus, jeopardizing the response to immunotherapies.^[^ ^60^ ^]^

**Figure 4:**
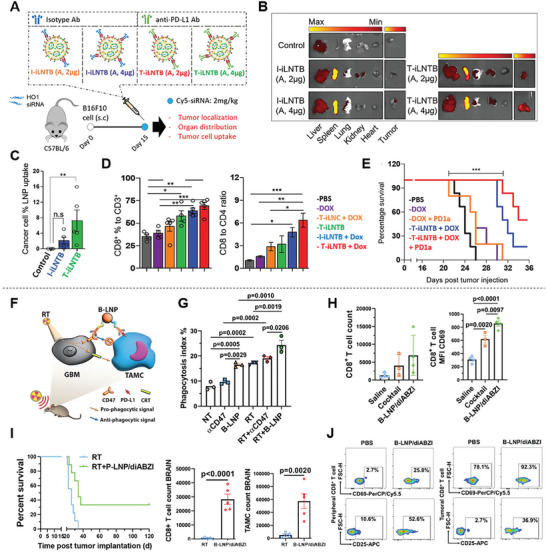
LNPs targeting tumor myeloid cells to enhance immunotherapy. A) Schematic illustration of the experimental design of the in vivo studies using LNPs with (T‐iLNTB) or without (I‐iLNTB) PD‐L1 targeting. B) PD‐L1‐targeted LNPs show increased tumor accumulation deduced from biodistribution images. C) Flow cytometry analysis reveals higher uptake of target LNPs (T‐iLNTB) in tumor cells when compared with the control and I‐iLNTB groups. Combination therapy using PD‐L1‐targeted LNPs (T‐iLNTB) with doxorubicin (DOX) D) enhances the transition from “cold” into “hot” tumor by improving the recruitment of cytotoxic CD8+ T cells and E) boosts the response to ICI (anti‐PD1a antibody), resulting in increased survival rates in vivo. F) Schematic illustration of the in vivo bridging effect of LNPs (B‐LNP/diABZI) engaging tumor‐associated myeloid cells (TAMCs) with glioblastoma (GBM) cancer cells. G) TAMC phagocytosis of CT‐2A glioma cells treated with anti‐CD47 antibody (free or B‐LNP‐conjugated form) at 10 µg mL^–1^ for 4 h at 37 °C and in the presence or absence of radiation therapy (RT). H) Quantification of CD8+ T cell accumulation at the tumor site after exposure to LNPs. I) B‐LNP/diABZI treatment in combination with RT reshapes the immune microenvironment and potentiates the survival of animals with murine glioma by recruitment of CD8+ T cells and TAMCs. J) Flow cytometry data showing the activation of glioblastoma patient‐derived T cells after exposure to antihuman CD47/PD‐L1‐functionalized‐B‐LNP/diABZI, with higher CD25 and CD69 expression. (A–E) Adapted under the terms of the Creative Commons Attribution‐NonCommercial license.^[^ ^61^ ^]^ Copyright 2022, Yong et al. (F–J) Adapted under the terms of the Creative Commons Attribution 4.0 International license.^[^ ^62^ ^]^ Copyright 2023, Zhang et al.

Therefore, tumor myeloid cell targeting might be relevant to enhance immunotherapy efficiency. For example, Yong et al. used ionizable LNPs, loaded with siRNA against heme oxygenase‐1 (HO1) and decorated with anti‐programmed death ligand 1 (PD‐L1) antibody, for a combinatorial therapeutic strategy aiming at enhancing chemotherapy and inducing immunological reprogramming on tumor myeloid cells and cancer cells (**Figure** **4**A).^[^ ^61^ ^]^ The accumulation of the LNPs at the tumor site and uptake by tumor cells (Figure 4B,C) by the silencing of HO1 (a pro‐tumorigenic enzyme responsible for chemoresistance onset and polarization of M1/M2 macrophages) efficiently sensitized cancer cells to chemo‐immunotherapy and promoted the recruitment of effector T cells at the tumor site (Figure 4D). This resulted in higher survival rates, i.e., 50% for the triple therapy T‐iLNTB+Dox+PD1a group versus 16.6% for the dual therapy T‐iLNTB+Dox group at day 36 (Figure 4E).^[^ ^61^ ^]^ In another study, Zhang et al. designed LNPs to engage tumor‐associated MDSCs and glioblastoma cells via anti‐CD47/PD‐L1 dual ligation (Figure 4F).^[^ ^62^ ^]^ The findings of the study showed that the simultaneous blockage of CD47 and PD‐L1 increased MDSC phagocytic activity (Figure 4G). Additionally, the loading of LNPs with diamidobenzimidazole (diABZI) resulted in the transcriptomic and metabolic switch of MDSCs into antitumor effectors, thus inducing CD8+ T cell infiltration and activation in brain tumors in vivo (Figure 4H). diABZI is a small drug molecule that serves as a nonnucleotidyl agonist for the activation of the stimulator of interferon (IFN) genes (STING). The combination of this approach with radiotherapy resulted in TME reshaping, which led to tumor regression and promoted immunological memory against glioma (Figure 4I). The activation of patient‐derived T cells was also demonstrated (Figure 4J).^[^ ^62^ ^]^

**Figure 5:**
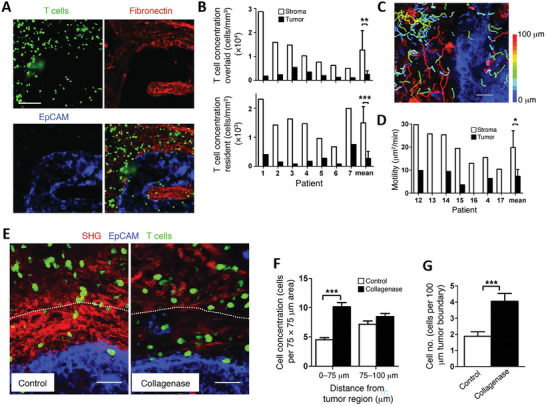
ECM organization and architecture determine T cell localization within the TME. A) Representative images of preactivated T cells (green) added to a human lung tumor slice stained for stromal compartment (fibronectin – red) and epithelial cells (epithelial cellular adhesion molecule, EpCAM – blue) and B) respective quantification analyses showing preferential accumulation of T cells within the stroma. C) Representative image and D) respective quantification of T cell motility within the TME reveal higher motion within the stroma. E) Representative images of tumor tissues before and after collagenase treatment identifying collagen (second harmonic generation, SHG – red) tumor cells (EpCAM – blue) and T cells (green). F) Number of T cells in 75 µm × 75 µm zones adjacent to tumor cell regions. G) Number of T cells in contact with peripheral cancer cells along the tumor–stroma boundary. Adapted with permission.^[^ ^69^ ^]^ Copyright 2012, American Society for Clinical Investigation.

Immune cells that are diffusely infiltrated within the tumor account for better prognosis compared to cells being confined to the perivascular space acting as bystanders. Many cancer patients fail to respond to immunotherapy owing to the limited number of effector T cells that reaches the tumor mass, which is a key factor for therapeutic success.^[^ ^63^ ^]^ The degree of infiltration of effector CD8+ T cells within the TME is categorized as “hot” or “cold”, characterized by a high or low infiltration of T cells, respectively.^[^ ^64^ ^]^ “Cold” tumors are typically responsible for the resistant phenotype observed among solid tumors.^[^ ^64^ ^]^ Several studies have shown that the presence of CD8+ and CD4+ T cells at the tumor sites are associated with good clinical outcome.^[^ ^65^ ^]^ In particular, it has been shown that for the successful eradication of tumor cells, CD8+ T cells need to engage with the target for an extended time, i.e., 5 min in vitro and 30 min to 2 h in vivo.^[^ ^66^ ^]^ In addition, Weigelin et al. reported that multiple contacts between T cells and tumor cells were required to efficiently kill the tumor cells.^[^ ^67^ ^]^ More specifically, using live‐cell microscopy, the authors demonstrated in vitro and in vivo, that tumor cells needed to be targeted at least three times by T cells within 3 h to be eradicated. In general, in 80% of the cases, the contact was achieved by multiple T cells rather than one. Overall, the study showed that single T cells attacks were insufficient to eliminate a tumor cell in large tumors. CD4+ T cells can either work as helper cells by secreting inflammatory cytokines or kill tumors cells via ligation of FasL and tumor necrosis factor‐related apoptosis‐inducing ligand pathways.^[^ ^66^ ^]^ Tumor cell killing via these pathways also requires lasting engagement (hours) with the tumor cells. In contrast, for their helper function, CD4+ cells do not necessarily need to be in contact with the tumor cells but they need to be in the proximity of cancer cells. Similarly to other cells, T cells use collagen aligned fibers to migrate along the ECM.^[^ ^68^ ^]^ It is assumed that while trafficking along these fibers at high speed, T cells are unable to engage with tumor cells as efficiently as when they move slower. Therefore, collagen remodeling and alignment, typical from desmoplastic phenotypes, may highjack T cell patrol and clearance of the tumor by retaining the T cells in the stromal compartment. To overcome the hindrance of ECM in antitumor immunity, research has focused on modulating the ECM features to enhance immunotherapy. In this field, it has been shown that the treatment of human lung tumors with collagenase, causing matrix reduction, significantly increases the number of T cells in contact with the tumor cells (**Figure** **5**).^[^ ^69^ ^]^

### 4.2 Safety

Similar to other types of cancer treatments, immunotherapy may lead to the occurrence of side effects. However, in this case, immune‐related adverse events (irAEs) are a form of newly developed autoimmune diseases that can affect any organ in the body.^[^ ^70^ ^]^ These irAEs are known to occur during immune checkpoint inhibition and pose a serious threat to the health of the patients.^[^ ^70^ ^]^ In 2022, Yan et al. have reported that although the toxicity profiles of ICIs seem more favorable than chemotherapy, irAEs occur more frequently and can potentially evolve into severe complications, ultimately leading to the discontinuation of the treatment or even death.^[^ ^71^ ^]^ The study alerts the occurrence of irAES as a result of using ICIs for advanced lung cancer treatment. The frequently reported adverse effects are pneumonitis and colitis or those associated with higher mortality (i.e., myocarditis, pneumonitis, and hepatitis). Therefore, it is advised that patients pursing immunotherapy are to be closely monitored by clinicians.^[^ ^71^ ^]^

In fact, off‐target immune toxicity remains a challenge to be solved. To circumvent this obstacle, specific biomarkers expressed by immune cells must be identified to determine those that can offer more accurate immune responses. As an alternative, direct intratumoral injection of immunotherapies has been described as a feasible methodology to reduce the risk of irAEs.^[^ ^72^ ^]^ Preclinical studies have shown that the local injection of immunomodulatory products stimulates the release of type I IFNs and enhances tumor antigen presentation on immune cells. This effect helps generate a stronger antitumor immune response, with the prospect to turn “cold” tumors into “hot” tumors.^[^ ^72^ ^]^ The administration of immune modulators directly into the tumor includes the use of nucleic acids,^[^ ^73^ ^]^ proteins,^[^ ^74^ ^]^ small molecules,^[^ ^75^ ^]^ and cell therapies.^[^ ^76^ ^]^

**Figure 6:**
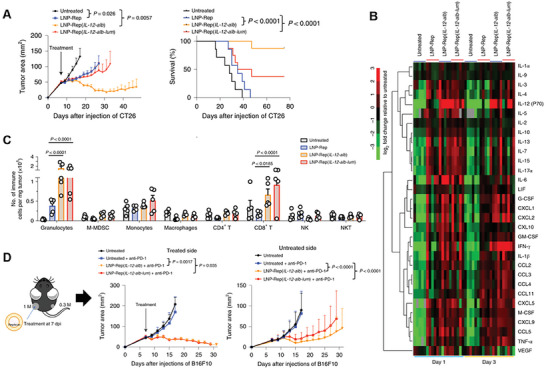
LNP intratumoral administration enhances systemic immunotherapy. A) Tumor regression and (increased) survival data following local tumor treatment with LNPs carrying IL‐12 replicon (LNP‐Rep). B) TME cytokine upregulation after local administration of LNP‐Rep. C) Immune cell quantification at the tumor site reveals enrichment of granulocytes and CD8+ T cells. D) Local injection of LNP‐Rep provides systemic antitumor effect, leading to the reduction of nontreated tumors. Adapted under the terms of the Creative Commons Attribution 4.0 International license.^[^ ^79^ ^]^ Copyright 2020, Li et al.

Liu et al. reported the intratumoral delivery of IL‐12 and IL‐27 mRNA using LNPs for treating melanoma in B16F10 mice.^[^ ^77^ ^]^ The results showed that the local administration of mRNAs induced an effective infiltration of immune effector cells, including natural killer (NK) and CD8+ T cells, into the tumor, while reducing systemic toxicity effects. In a different study, Hewitt et al. reported the development of an LNP formulation to deliver IL‐12 mRNA (MEDI1191)‐based therapy through intratumoral injection in vivo using mice models and ex vivo using patient tumor slice cultures.^[^ ^78^ ^]^ This work showed that a single dose of mIL12 mRNA–LNPs induced tumor regression in multiple syngeneic mice models. Additionally, nearly all animals examined did not grow tumors when exposed to the same tumor type for the second time, suggesting the development of an immune memory response. A single injection of mIL12 mRNA–LNPs induced complete regression of both the treated and untreated distal tumors in 3 out of the 20 animals examined and significantly improved overall survival. The antitumor effect of the mIL12 mRNA–LNPs, which was dose‐dependent, was increased in combination with anti‐PD‐L1. The findings of the study supported the use of MEDI1191 in patients with both superficial and deep‐seated solid tumors. Intratumoral administration of MEDI1191 is currently under clinical trials Phase 1 for treating solid tumors in combination with durvalumab, an anti‐PD‐L1 drug (NCT03946800).^[^ ^78^ ^]^ A similar approach using LNPs carrying a replicon (LNP‐Rep) encoding for IL‐12 revealed that a single local injection in vivo could determine the rejection of large established tumors (**Figure** **6**A).^[^ ^79^ ^]^ All LNP‐Rep treatments induced the upregulation of inflammatory cytokines and chemokines at the TME with the IL‐12‐encoding replicons eliciting IFN‐γ production (Figure 6B). Such high levels of intratumoral IL‐12 correlated with cytokine dissemination in the blood and systemic IFN‐γ production and toxicity. Therefore, to help contain systemic toxicity, by retaining the cytokines within the TME via the lumican domain, IL‐12 fusion to the ECM‐binding protein lumican was performed. Additionally, a large influx of granulocytes and CD8+ T cells at the TME were observed after LNP‐Rep treatment (Figure 6C). Furthermore, the locally injected LNP‐Rep therapy induced the regression of distal untreated tumors and eliminated metastases (Figure 6D).^[^ ^79^ ^]^

Besides the issues inherent to immunotherapy itself, the use of lipid‐based nanoparticles as delivery vehicles might contribute to immunogenicity.^[^ ^80^ ^]^ Ndeupen et al. demonstrated that LNP–mRNA vaccines were highly inflammatory in mice models.^[^ ^80^ ^]^ After administration of LNPs via intradermal, intramuscular, or intranasal injection, severe inflammatory responses were registered owing to considerable neutrophil infiltration, production of various inflammatory cytokines, and activation of diverse inflammatory pathways, resulting in high mortality rate of the animals.^[^ ^80^ ^]^ The use of cationic lipids in LNP formulations for effective encapsulation of negatively charged nucleic acids, through electrostatic interactions, has shown relatively toxic events in vivo.^[^ ^81^ ^]^ Kedmi et al. showed that positively charged LNPs caused a dramatic pro‐inflammatory response, 10–75‐fold higher compared to neutral and negatively charged nanoparticles. The work showed that cationic lipids activated Toll‐receptor 4, expressed on leucocytes, in a specific manner.^[^ ^81^ ^]^ To address this issue, ionizable cationic lipids have been successfully developed and used in clinical products, i.e., Onpattro and the two COVID‐19 vaccines developed by Pfizer‐BioNTech and Moderna. As reported in the literature, although the ionizable cationic lipids displayed improved efficacy over permanently charged lipids, individuals subjected to LNP–mRNA vaccines displayed side effects such as local pain, fever, swelling, and systemic inflammatory responses.^[^ ^82^ ^]^

In addition, PEGylated lipids, which are typically used to increase the stability and circulation half‐life of LNPs, induce the secretion of anti‐PEG antibodies that may cause hypersensitivity reactions as well as the activation of the complement system.^[^ ^83^ ^]^ This increases the risk of undesirable clearance of the nanoparticles, reducing the antitumor efficacy.^[^ ^83^ ^]^ Nevertheless, PEG has been widely employed to coat the surface of lipid‐based nanotherapeutics used in clinic, including Doxil, Onivyde, and Onpattro.^[^ ^84^ ^]^ Several studies have focused on the role of PEG coating on nanoparticle interactions with immune cells, and their outcomes can be leveraged to improve the formulation of LNPs and maximize their association with the desired immune system components. For instance, varying PEG architecture influenced nanoparticle association with monocytes and ultimately their biological behavior in blood.^[^ ^85^ ^]^ In addition, PEG‐coated nanoparticles incubated ex vivo with plasma samples from various donors formed personalized coronas, which correlated with the blood immune cell interactions of nanoparticles.^[^ ^86^ ^]^ Reports have also suggested the use of PEG alternatives, such as polysarcosine, which have similar physicochemical properties to PEG but are less immunogenic, and thus may potentially replace PEG in LNP‐mediated mRNA delivery.^[^ ^87^ ^]^

### 4.3 Efficiency

Translation issues regarding the use of lipid‐based nanoparticles for onco‐immunotherapy also result because the in vivo mice models, as used in most studies, do not accurately replicate the human oncologic physiology.^[^ ^88^ ^]^ Despite the limitations and controversies associated with their use, mice still represent the gold standard for experimentation, due to their inherent advantage over standard in vitro models. In 2021, Hassett et al. described the influence of LNP biophysical properties on mRNA vaccine immunogenicity using mice and nonhuman primates (NHPs) as animal models.^[^ ^89^ ^]^ The findings showed that between the different formulations of LNPs with various sizes (60–200 nm) examined, the smaller LNPs were significantly less immunogenic for BALB/C mice than the larger LNPs but all LNPs examined, irrespective of their size, displayed a robust immune response in NHPs.^[^ ^89^ ^]^ These contrasting results emphasize the concerns about selecting the appropriate animal model and the potential translational limitations to humans owing to interspecies physiological differences. Lam et al. showed how the optimization of LNP parameters such as size (50–60 nm versus the optimal 70–80 nm determined for rodents) and amount of PEG coating (almost double PEG‐conjugated lipid when compared to the formulations used in rodents) for LNP application in NHPs resulted in an eightfold increase in protein expression after IV injection of mRNA–LNPs.^[^ ^90^ ^]^ These findings underline the need to overcome the clinical challenges imposed by the use of inappropriate models, which increase the difficulty of accomplishing relevant therapeutic outcomes at tolerable doses in larger species.^[^ ^90^ ^]^ Although NHPs are generally considered a better predictive model, ethical and economic issues restrict their use. Hence, LNP compositions have been historically optimized in rodent models. In response to this obstacle, several mice models have been developed to improve the therapeutic potential translation to humans, including humanized mouse models.^[^ ^91^ ^]^ Nevertheless, humanized models require the immunodeficient mice to be humanized with a human immune system, which requires engraftment of peripheral blood mononuclear cells and has yet to be optimized.^[^ ^92^ ^]^ In 2022, Hatit et al. reported that the delivery of mRNA using 89 chemically distinct LNPs in humanized, primatized, and murinized livers resulted in species‐dependent responses to LNPs, including mRNA translation and endocytosis.^[^ ^93^ ^]^ The data generated in the study propose that for individual LNP studies, the efficacy data obtained using humanized mice supersede the data obtained from wild‐type mice, whereas the safety data from wild‐type mice supersede the safety data obtained from humanized mice. Yet, two limitations of this work are acknowledged: (i) the use of humanized and primatized mice as representations for humans and NHPs and (ii) the use of LNPs that are likely to target hepatocytes. Therefore, Hatit et al. hypothesized that other LNPs and different cell types might result in significantly different responses.^[^ ^93^ ^]^ Despite advances achieved using this model as a preclinical bridge to understand the immune functions of novel anticancer vaccines, humanized models remain limited by low engraftment rates, suboptimal development of lymphatic organs, and variability in major histocompatibility complex antigens.^[^ ^92^, ^94^ ^]^ In contrast, genetically engineered mice that can spontaneously develop cancer are relevant models with a more similar TME to that found in humans. For example, HPV transgenic mice have immune signatures of the HPV similar to that of squamous cell carcinoma.^[^ ^95^ ^]^ This model offers a better alternative to evaluate the infiltration of immune cells after treatment with nanoformulations. Nevertheless, whether the immunotherapy efficacy can be safely translated to patients remains to be carefully evaluated.

## 5 Perspectives

As previously described in the literature, the composition of the TME may represent a hindrance to the success of immunotherapy.^[^ ^96^ ^]^ The desmoplastic ECM, which consists most of the solid tumor mass, restricts T cell infiltration across the tumor to target the malignant T cells.^[^ ^55a^ ^]^ Numerous therapeutic approaches are currently under clinical trial evaluation for that purpose. For instance, Simtuzumab, a monoclonal antibody (mAB) directed against lysyl oxidase‐like 2 (LOXL2) that catalyzes the cross‐linking of collagen and elastin, contributes to fibrotic ECM stabilization.^[^ ^97^ ^]^ In particular, the inhibition of LOXL2 expression is known to reduce the number of activated fibroblasts, decrease ECM deposition, inhibit angiogenesis, and prevent tumor cell invasion and the consequent risk of metastasis.^[^ ^98^ ^]^ However, the use of Simtuzumab in a Phase 2 study (clinicaltrials.gov identifier: NCT01769196) in patients with idiopathic pulmonary fibrosis and colorectal and pancreatic cancers was not supported by clinical benefit and was therefore discontinued. Several molecular mechanisms contribute to the alterations observed at the tumorigenic ECM. In addition, the activation and involvement of each pathway in the tumor stiffening process are stage‐ and disease‐dependent. Therefore, a combinatorial therapeutic approach is more likely to succeed. Studies based on the use of mAB delivery were performed to normalize the ECM. The combination of trastuzumab (an mAB targeting human epidermal growth factor receptor 2 in breast cancer) and hyaluronidase‐osyk, commercially available as Herceptin Hylecta, has shown that the use of the latter facilitates the subcutaneous dispersion of the antibody by inducing hyaluronan degradation.^[^ ^8^ ^]^ Moreover, Pamrevlumab, a commercially available mAB against connective tissue growth factor (CTGF), is currently under Phase 3 study as a neoadjuvant for chemotherapy in pancreatic cancer (NCT03941093).

**Figure 7:**
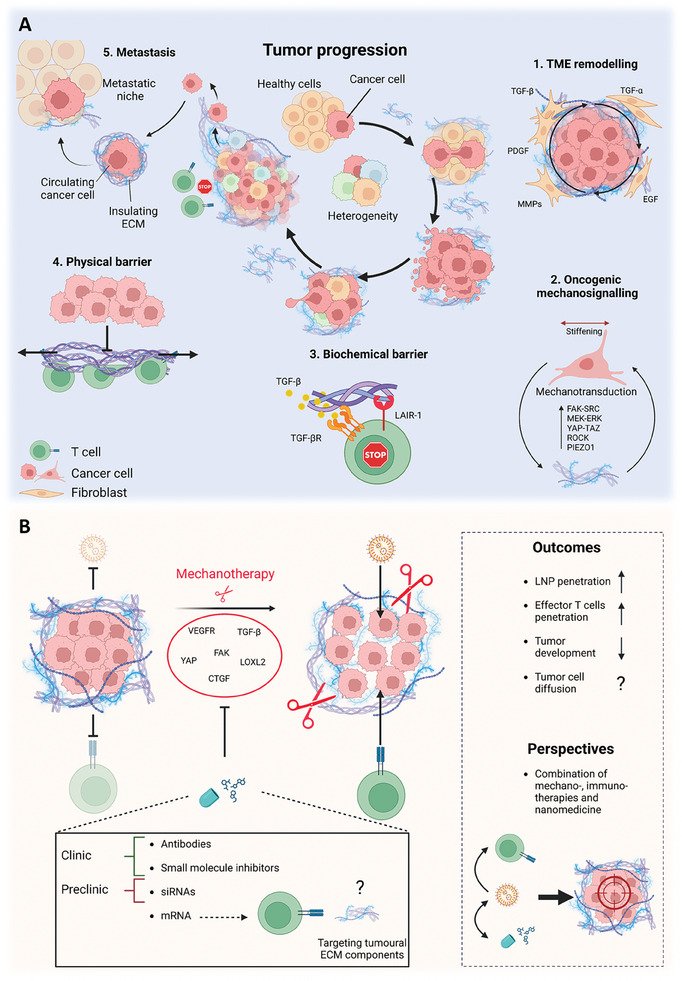
ECM deposition and remodeling implications for tumor progression and immunotherapy resistance. A) In a simplified view, the deregulated secretion of various factors (e.g., TGF‐β, PDGF, EGF) at the preneoplastic site contributes to ECM deposition and remodeling in a process that promotes tumorigenicity and sustains the deposition of aberrant ECM desmoplasia. These events promote the formation of a TME that fuels cancer cell survival and tumor progression (1). The ECM‐driven oncogenic mechanosignaling leads to the activation of several pathways involved in the transcription of genes that regulate cell proliferation, survival, migration, and immune system escape, thereby further enhancing ECM remodeling and deposition in a cancer progression‐promoting loop (2). The desmoplastic ECM can also create a biochemical barrier as described for collagens, which act as ligands for ICI receptor LAIR‐1. Binding of LAIR‐1 collagens inhibits T and NK cell functions (3). Besides, the dense layer of ECM surrounding the tumor creates a physical obstacle limiting the diffusion of T cells, which move along the aligned fibers, throughout the TME and thus restricting their effective tumor killing activity (4). Altogether, the occurrence of desmoplasia contributes to stimulating cancer cells into a more aggressive phenotype, with enhanced migratory and invasive properties, as well as immune surveillance escape capacity, which potentially leads to the occurrence of metastasis (5). B) Mechanotherapy approaches aiming to modulate tissue stiffness by loosening the desmoplastic ECM via the downregulation/inhibition of different proteins, such as TGF‐β, YAP, CTGF, LOXL2, and VEGFR. This is expected to enhance nanoparticle delivery and T cell infiltration at the tumor site, ultimately enhancing the efficacy of immunotherapies. Created with BioRender.com.

Preneoplastic tissues exhibit features of desmoplastic/fibrotic ECM that contribute to malignant transformation and tumor progression (**Figure** **7**A). The excessive deposition and remodeling of ECM proteins and subsequent tumor stiffening occur in response to the aberrant expression/secretion of profibrotic proteins/factors at the TME and are maintained during TME remodeling and tumor progression (Figure 7A‐1). The profibrotic proteins/factors at the TME include transforming growth factor (TGF)‐β, TGF‐α, platelet‐derived growth factor (PDGF), and epidermal growth factor (EGF).^[^ ^49^ ^]^ Furthermore, tissue mechanosensing is related to ECM configuration. Several mechanobiological pathways are deregulated in response to ECM stiffening and, in turn, further sustain ECM remodeling and deposition, in a bidirectional interplay defined as “oncogenic mechanosignaling” that promotes tumor development (Figure 7A‐2). For instance, it has been reported that yes‐associated protein (YAP)‐transcriptional coactivator with PDZ‐binding motif (TAZ) transcriptional activity is able to inhibit the pro‐inflammatory cyclic GMP–AMP synthase (cGAS)‐STING pathway, thus contributing to immune system suppression and tumor establishment.^[^ ^99^ ^]^ The desmoplastic ECM can also create a biochemical barrier by interfering with signaling pathways, which lowers the immune surveillance at the TME. For instance, collagen fibers can bind to leukocyte associated immunoglobulin‐like receptor‐1 (LAIR‐1) and reduce NK and T cell activation (Figure 7A‐3).^[^ ^100^ ^]^ LAIR‐1 signaling has been shown to lead to T cell exhaustion, rendering lung tumors resistant to programmed cell death protein 1 (PD1)/PD‐L1 therapy.^[^ ^101^ ^]^ Likewise, the fibrotic ECM, physically confining the tumor mass, functions as a physical barrier that affects the infiltration of cytotoxic T cells and the delivery of chemotherapeutics (Figure 7A‐4). At later stages of tumor development, the excessive remodeling of desmoplastic ECM promotes cancer progression through diverse signaling pathways, including and nonrestricted to TGF‐β, Wnt‐β‐catenin, and Notch, that lead to EMT and fuel metastasis (Figure 7A‐5).^[^ ^102^ ^]^ In addition, the nature of the ECM at the primary and metastatic tumor sites is comparable. Similar remodeling and proteolytic events have been observed at the tumor tissues and premetastatic niche.^[^ ^103^ ^]^ Finally, invasive tumor cells can also exploit ECM components to shield themselves from shear stress during circulation or to escape immune surveillance.^[^ ^103^ ^]^ Hence, establishing a therapy that can normalize tumor ECM would reduce pro‐tumorigenic signaling at the TME, while promoting infiltration of effector immune cells and nanotherapy delivery at the tumor site (Figure 7B). Herein, the downregulation/inhibition of different proteins or soluble cues responsible for activating cancer cells and CAFs, inducing excessive ECM deposition and remodeling, might be interesting targets of study to reduce the tumorigenic desmoplastic phenotype impeding T cell infiltration at the TME. These would include TGF‐β,^[^ ^51^, ^54^ ^]^ YAP,^[^ ^104^ ^]^ CTGF,^[^ ^105^ ^]^ LOXL2,^[^ ^106^ ^]^ and vascular endothelial growth factor receptor (VEGFR).^[^ ^107^ ^]^ Furthermore, investigation of the tumor ECM proteasome, e.g., receptors mediating cell–ECM interactions or ECM domains tightly associated with cell transmembrane receptors, may lead to the discovery of novel targets to instruct the immune system cells against tumors in time and stage‐related intervention, using LNP–mRNA formulations.

**Figure 8:**
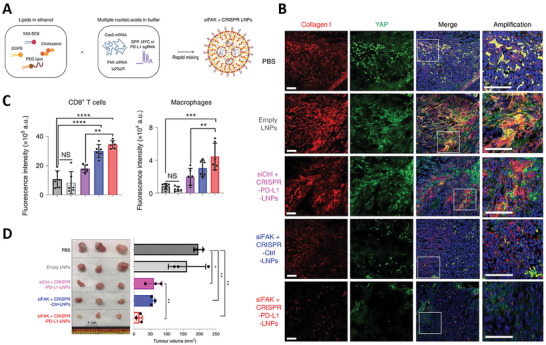
Modulating the ECM and tumor stiffness boost immunotherapy. A) Schematic representation of loading of FAK siRNA, Cas9 mRNA, and sgRNA into 5A2‐SC8 LNPs. B) Representative 3D construction of immunofluorescence of collagen I and YAP in fixed tumor tissues after therapy for 30 days in vivo. C) Quantification of infiltration of macrophages and CD8+ T cells at the TME. (D) Excised tumor size measurements show in vivo therapeutic efficacy. Adapted under the terms of the Creative Commons Attribution 4.0 International license.^[^ ^108^ ^]^ Copyright 2022, Zhang et al.

In this context, Zhang et al. showed that the accumulation of CRISPR/Cas9 in solid tumors via LNPs was enhanced by targeting focal adhesion kinases (FAKs) (**Figure** **8**A).^[^ ^108^ ^]^ The study showed that the codelivery of anti‐FAK siRNA, Cas9 mRNA, and single guide RNA (sgRNA) (directed against PD‐L1) via LNPs, after repeated administrations, reduced collagen deposition (Figure 8B) and ECM stiffness. The use of LNPs loaded with such mix of cargos consequently (i) enhanced the delivery of LNPs, (ii) enhanced the infiltration of T cells and macrophages at the TME (Figure 8C), and (3) inhibited tumor growth (Figure 8D) in comparison to the use of nanoparticles carrying the sole gene editing construct against PD‐L1.^[^ ^108^ ^]^ Although the application of antifibrotic therapies may be promising in terms of therapeutic intervention, careful considerations should be made on whether the loosening of the ECM could cause excessive tumor cell evasion or leaking from the tumor site, with eventual increased risk of metastasis. Few studies have demonstrated that a less dense and soft ECM can promote tumor immunogenicity.^[^ ^109^ ^]^ Nevertheless, although the disruption of tumor ECM facilitates interstitial transport of therapeutics and immune cells to the tumor, it might also lower the barriers to tumor cell metastasis, with unclear clinical outcomes.

Along with the complexity of the 3D structure and spatiotemporal development of tumors, understanding their ability to trigger the activation of specific pro‐tumorigenic pathways at specific stages of disease progression requires the use of advanced cellular models and an understanding of the molecular players driving tumor progression. An ideal platform for evaluating onco‐immunotherapies entails the coculture of the diverse cells constituting the TME, thus faithfully resembling—histologically and functionally—the in vivo tumor to generate accurate treatment responses. Recent advances in cell culturing models have highlighted organoid systems as promising platforms to access the efficacy of different oncology therapies.^[^ ^110^ ^]^ Cancer organoids are classified as multicellular, self‐organizing avatars that resemble tumor physiology, offering advantages over traditional cell culture methodologies.^[^ ^110^, ^111^ ^]^ Additionally, organoids prepared directly from patient tumor tissue overcome the limitation that some diseases are not replicable in animal models. While these models offer great promise in the field of personalized therapy, they can also be used to identify a spectrum of effective drugs against different tumors. Alternatively, extrusion‐based 3D cellular bioprinting has been employed to generate highly reproducible organoids to facilitate drug screening.^[^ ^112^ ^]^ Nevertheless, limitations, such as scale up issues, remain to be addressed. Recent, significant progress has been made in the field using organoids. In 2022, the multinational pharmaceutical company Roche implemented the use of these mini organs in the field of drug discovery.^[^ ^113^ ^]^ Given the success achieved in the field of organoids and the possibility to integrate TIME modeling, it is appealing to consider the use of such models to evaluate LNP‐based cancer immunotherapy. This would likely lead to better therapeutic response predictions. Nevertheless, few studies have reported the use of these unique models for immunotherapy evaluations. For instance, Neal et al. described a methodology to prepare patient‐derived organoids with preserved original tumor T cell receptors and immune check point blockage, as confirmed by single‐cell transcriptome.^[^ ^114^ ^]^ In a different study, Tsai et al. reported the use of organoids in coculture with CAFs and T cells to recreate patient‐matched organotypic models of pancreatic cancer, suitable for studying immune cell–tumor interactions.^[^ ^115^ ^]^

**Figure 9:**
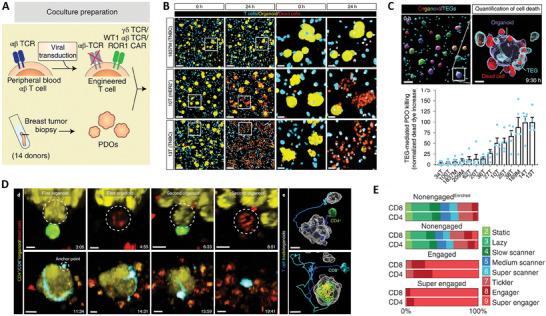
A) Schematic illustration of generation and coculture of engineered T cells (TEGs) with PDOs. B) 3D multispectral images of breast PDO cultures (yellow) with low (1837M), intermediate (10T), and high (13T) killing by TEGs (blue). C) 3D images of organoids and T cells; enlarged section shows the presence of dead cells (red) in a single organoid (transparent purple rendering) and TEGs (transparent blue rendering). Quantification of organoid death derived after coculture with TEGs for 24 h. PDOs from 14 patients were used. D) Representative images of CD4+ TEG killing a tumor cell in an organoid and a second tumor cell in a neighboring organoid (upper), and CD8+ TEG killing a complete organoid over 11 h (lower). E) Distribution of the nine behavioral signatures identified for TEG populations isolated after 6 h of coculture with 13T PDOs. Adapted under the terms of the Creative Commons Attribution 4.0 International license.^[^ ^116^ ^]^ Copyright 2022, Dekkers et al.

Furthermore, Dekkers et al. described the development of an organotypic model using patient‐derived solid tumor organoids (PDOs) and engineered T cells (TEGs) (**Figure** **9**A) to study their interaction by imaging (Figure 9B–D) and transcriptomics (Figure 9E), identifying behavior‐specific gene signatures expressed by highly engaging killer TEGs. Through a complex analysis, the study unveils that killing activity of TEGs is primed by IFN‐β. These findings may support the optimization of personalized tumor‐targeting immunotherapies (Figure 9).^[^ ^116^ ^]^ Despite the substantial advances made in the field of organoids, orchestrated, multidisciplinary collaborative work is required for these models to become a reality in patient cancer healthcare. This progress combined with the permission granted by the FDA (through the FDA Modernization Act 2.0) to use certain alternatives to animal testing (i.e., cell‐based assays and computer models) to assess drugs before proceeding to administration to humans^[^ ^117^ ^]^ should encourage researchers and pharmaceutical industries to join efforts on the pursuit for better healthcare solutions for anticancer immunotherapies, including the development of standardized animal‐free robust in vitro models.

Nevertheless, to the best of our knowledge, despite the advancements achieved in the field of organoids, studies on the interaction of lipid‐based nanoparticles with these advanced tumor models are yet to be reported, with simple 3D spheroids models being used instead.^[^ ^118^, ^119^ ^]^

## 6 Concluding Remarks

The advances achieved in the field of immunotherapy hold promise for the treatment of solid tumors. During the last few years, significant progress in the field of nanotechnology has allowed the development of lipid‐based nanoparticles suitable for clinical application including cancer therapy. Since then, research efforts have focused on developing distinctive lipid nanoparticles to instruct the immune system to eliminate solid tumors rather than directly targeting the tumor cells. In comparison to other types of anticancer nanoparticle‐based treatments, an advantage of this lipid nanoparticle‐based approach is the unique property of lipid‐based immunotherapy to elicit a response in both primary and metastatic tumors owing to the stimulation of immune memory. Proof of concept for the treatment of metastasis has been provided in studies in which bilateral tumors are grown in the same animal, followed by local treatment of only one of the tumors with LNPs against specific cancer markers.^[^ ^78^, ^79^ ^]^ The use of these nanoparticles induces a suppressive immune response against the tumor at the primary site of injection and against cancer cells at a distal site, demonstrating effective stimulation of the immune system, likely against metastatic sites. Although these experiments provide valuable outcomes on how the immune system can retain its immunogenic action and react against distant tumors, they do not mimic the complex process of metastasis in patients, and particularly the genetic variability that metastatic cells acquire to adapt and survive to a new environment once they evade the primary tumor. Using a different approach, based on rechallenging experiments, mice are first vaccinated with lymph node‐targeting LNPs and then subjected to tumor inoculation to monitor tumor growth after immunization.^[^ ^120^ ^]^ Using this strategy, it has been shown that specific LNP formulations have the potential to enhance immune memory. Nevertheless, several challenges remain, and limitations need to be overcome in regard to the tumor models applied at preclinical stage, as well as therapeutic issues concerning patients’ eligibility and the occurrence of mild‐to‐severe side effects upon administration. Although few studies have shown promising results, many of them are based on the intratumoral administration of lipid‐based immunotherapies, which is unsuitable for many solid tumors.

The emergence of patient‐derived organoids, which have recently become a focal point in cancer research and are projected to become a leading model for preclinical investigations in the near future, holds the potential to enhance the histological characterization of tissues from various cancers, deepen the understanding of the molecular pathways involved in cancer cell development, and facilitate the identification of novel, unique, and more effective targets for tumors. In addition, the incorporation of immune cells into organoids offers new opportunities for using these advanced models to evaluate immunotherapies. Although, even when using organoids, the composition and architecture of the TME (including the interplay between different cell types and the existence of a desmoplastic ECM) need to be addressed for the model to be predictive. The tumorigenic ECM, being the major component of the TME, is largely responsible for impairing the diffusion of T cells and nanoparticles at the tumor site as well as for fueling tumor progression by stimulating cancer cells into more aggressive phenotypes. Therefore, exploiting the use of mechanotherapy approaches, aiming at normalizing the occurrence of fibrotic ECM at the TME may benefit the outcome of lipid‐based immunotherapy. This, in turn, would ultimately expand the repertoire of LNP formulations and cargos available for therapeutic screening assessment and enable improved patient stratification into more effective and diverse cohorts. Altogether, lipid‐based immunotherapy has the potential to revolutionize cancer treatment.

## Conflict of Interest

The authors declare no conflict of interest.

## References

[1] L. Xu , X. Wang , Y. Liu , G. Yang , R. J. Falconer , C.‐X. Zhao , Adv. NanoBiomed Res. 2022, 2, 2100109.

[2] a) Y. Zong , Yi Lin , T. Wei , Q. Cheng , Adv. Mater. 2023, 2303261; 10.1002/adma.202303261 37196221

[3] Y. Zhu , R. Shen , I. Vuong , R. A. Reynolds , M. J. Shears , Z.‐C. Yao , Y. Hu , W. J. Cho , J. Kong , S. K. Reddy , S. C. Murphy , H.‐Q. Mao , Nat. Commun. 2022, 13, 4282. 35879315 10.1038/s41467-022-31993-y PMC9310361

[4] a) R. M. Haley , A. Chan , M. M. Billingsley , N. Gong , M. S. Padilla , E. H. Kim , H. Wang , D. Yin , K. J. Wangensteen , A. Tsourkas , M. J. Mitchell , ACS Appl. Mater. Interfaces 2023, 15, 21877; 37115558 10.1021/acsami.3c01501 PMC10727849

[5] R. Tenchov , R. A. Bird , A. Curtze , Q. Zhou , ACS Nano 2021, 23, 16982. 10.1021/acsnano.1c04996 34181394

[6] S. Qin , X. Tang , Y. Chen , K. Chen , Na Fan , W. Xiao , Q. Zheng , G. Li , Y. Teng , M. Wu , X. Song , Signal Transduction Targeted Ther 2022, 7, 166. 10.1038/s41392-022-01007-w PMC9123296 35597779

[7] X. Han , H. Zhang , K. Butowska , K. L. Swingle , M.‐G. Alameh , D. Weissman , M. J. Mitchell , Nat. Commun. 2021, 12, 7233. 34903741 10.1038/s41467-021-27493-0 PMC8668901

[8] A. M. Vargason , A. C. Anselmo , S. Mitragotri , Nat. Biomed. Eng. 2021, 5, 951. 33795852 10.1038/s41551-021-00698-w

[9] a) P. R. Cullis , M. J. Hope , Mol. Ther. 2017, 25, 1467; 28412170 10.1016/j.ymthe.2017.03.013 PMC5498813

[10] a) Q. Cheng , T. Wei , L. Farbiak , L. T. Johnson , S. A. Dilliard , D. J. Siegwart , Nat. Nanotechnol. 2020, 15, 313; 32251383 10.1038/s41565-020-0669-6 PMC7735425

[11] S. Zhen , Xu Li , Cancer Gene Ther 2020, 27, 515. 31676843 10.1038/s41417-019-0141-7

[12] H. Lv , S. Zhang , B. Wang , S. Cui , J. Yan , J. Controlled Release 2006, 114, 100. 10.1016/j.jconrel.2006.04.014 16831482

[13] C. R. Stadler , H. Bähr‐Mahmud , L. Celik , B. Hebich , A. S. Roth , R. P. Roth , K. Karikó , Ö. Türeci , U. Sahin , Nat. Med. 2017, 23, 815. 28604701 10.1038/nm.4356

[14] A. J. Barbier , A. Y. Jiang , P. Zhang , R. Wooster , D. G. Anderson , Nat. Biotechnol. 2022, 40, 840. 35534554 10.1038/s41587-022-01294-2

[15] M. Kaduri , M. Sela , S. Kagan , M. Poley , H. Abumanhal‐Masarweh , P. Mora‐Raimundo , A. Ouro , N. Dahan , D. Hershkovitz , J. Shklover , J. Shainsky‐Roitman , Y. Buganim , A. Schroeder , Sci. Adv. 2021, 7, eabj5435. 34613777 10.1126/sciadv.abj5435 PMC8494443

[16] a) S. M. Hoy , Drugs 2018, 78, 1625; 30251172 10.1007/s40265-018-0983-6

[17] A. Gabizon , D. Goren , Z. Fuks , Y. Barenholz , A. Dagan , A. Meshorer , Cancer Res 1983, 43, 4730. 6883331

[18] D. Rosenblum , N. Joshi , W. Tao , J. M. Karp , D. Peer , Nat. Commun. 2018, 9, 1410. 29650952 10.1038/s41467-018-03705-y PMC5897557

[19] Y. (.C.). Barenholz , J. Controlled Release 2012, 160, 117. 10.1016/j.jconrel.2012.03.020 22484195

[20] S. Fernandes , M. Cassani , S. Pagliari , P. Filipensky , F. Cavalieri , G. Forte , Curr. Med. Chem. 2020, 27, 7234. 32586245 10.2174/0929867327666200625151134

[21] F. C. Passero , D. Grapsa , K. N. Syrigos , M. W. Saif , Expert Rev. Anticancer Ther. 2016, 16, 697. 27219482 10.1080/14737140.2016.1192471

[22] M. Alfayez , H. Kantarjian , T. Kadia , F. Ravandi‐Kashani , N. Daver , Leuk. Lymphoma 2020, 61, 288. 31547736 10.1080/10428194.2019.1660970

[23] L. Beola , N. Iturrioz‐Rodríguez , C. Pucci , R. Bertorelli , G. Ciofani , ACS Nano 2023, 17, 18441. 37698887 10.1021/acsnano.3c06085 PMC10540267

[24] a) J. Zhang , Y. Lin , Z. Lin , Qi Wei , J. Qian , R. Ruan , X. Jiang , L. Hou , J. Song , J. Ding , H. Yang , Adv. Sci. 2022, 9, 2103444; 10.1002/advs.202103444 PMC8844476 34927373

[25] G. H. Petersen , S. K. Alzghari , W. Chee , S. S. Sankari , N. M. La‐Beck , J. Controlled Release 2016, 232, 255. 10.1016/j.jconrel.2016.04.028 27108612

[26] a) S. Wilhelm , A. J. Tavares , Q. Dai , S. Ohta , J. Audet , H. F. Dvorak , W. C. W. Chan , Nat. Rev. Mater. 2016, 1, 16014;

[27] U. Sahin , K. Karikó , Ö. Türeci , Nat. Rev. Drug Discovery 2014, 13, 759. 25233993 10.1038/nrd4278

[28] Bo Hu , L. Zhong , Y. Weng , L. Peng , Y. Huang , Y. Zhao , X.‐J. Liang , Signal. Transduction Targeted Ther. 2020, 5, 101. 10.1038/s41392-020-0207-x PMC7305320 32561705

[29] J. K. W. Lam , M. Y. T. Chow , Yu Zhang , S. W. S. Leung , Mol. Ther. Nucleic Acids 2015, 4, e252. 26372022 10.1038/mtna.2015.23 PMC4877448

[30] L. J. Scott , Drugs 2020, 80, 335. 32034693 10.1007/s40265-020-01269-0

[31] I. El Dika , H. Y. Lim , W. P. Yong , C.‐C. Lin , J.‐H. Yoon , M. Modiano , B. Freilich , H. J. Choi , T.‐Y. Chao , R. K. Kelley , J. Brown , J. Knox , B.‐Y. Ryoo , T. Yau , G. K. Abou‐Alfa , Oncologist 2019, 24, 747. 30598500 10.1634/theoncologist.2018-0838 PMC6656521

[32] B. B. Mendes , J. Conniot , A. Avital , D. Yao , X. Jiang , X. Zhou , N. Sharf‐Pauker , Y. Xiao , O. Adir , H. Liang , J. Shi , A. Schroeder , J. Conde , Nat. Rev. Methods Primers 2022, 2, 24. 35480987 10.1038/s43586-022-00104-y PMC9038125

[33] A. Da Silva Sanchez , K. Paunovska , A. Cristian , J. E. Dahlman , Hum. Gene Ther 2020, 31, 940. 32799680 10.1089/hum.2020.137 PMC7495921

[34] C. G. Perez‐Garcia , R. Diaz‐Trelles , J. B. Vega , Y. Bao , M. Sablad , P. Limphong , S. Chikamatsu , H. Yu , W. Taylor , P. P. Karmali , K. Tachikawa , P. Chivukula , Mol. Ther. Nucleic Acids 2022, 28, 87. 35356682 10.1016/j.omtn.2022.02.020 PMC8933640

[35] G. T. Szabó , A. J. Mahiny , I. Vlatkovic , Mol. Ther. 2022, 30, 1850. 35189345 10.1016/j.ymthe.2022.02.016 PMC8856755

[36] ClinicalTrials.gov, https://www.clinicaltrials.gov/, accessed: June, 2023.

[37] T. M. Raimondo , K. Reed , D. Shi , R. Langer , D. G. Anderson , Cell 2023, 186, 1535. 37059063 10.1016/j.cell.2023.02.031

[38] a) J. A. Marin‐Acevedo , E. O. Kimbrough , Y. Lou , J. Hematol. Oncol. 2021, 14, 45; 33741032 10.1186/s13045-021-01056-8 PMC7977302

[39] a) D. B. Johnson , C. A. Nebhan , J. J. Moslehi , J. M. Balko , Nat. Rev. Clin. Oncol. 2022, 19, 254; 35082367 10.1038/s41571-022-00600-w PMC8790946

[40] a) R. W. Jenkins , D. A. Barbie , K. T. Flaherty , Br. J. Cancer 2018, 118, 9; 29319049 10.1038/bjc.2017.434 PMC5765236

[41] P. Karmacharya , B. R. Patil , J. O. Kim , J. Pharm. Invest. 2022, 52, 415. 10.1007/s40005-022-00569-9 PMC8960215 35369363

[42] a) J. G. Rurik , I. Tombácz , A. Yadegari , P. O. Méndez Fernández , S. V. Shewale , L. Li , T. Kimura , O. Y. Soliman , T. E. Papp , Y. K. Tam , B. L. Mui , S. M. Albelda , E. Puré , C. H. June , H. Aghajanian , D. Weissman , H. Parhiz , J. A. Epstein , Science 2022, 375, 91; 34990237 10.1126/science.abm0594 PMC9983611

[43] A. Michels , N. Ho , C. J. Buchholz , Mol. Ther. 2022, 30, 2401. 35598048 10.1016/j.ymthe.2022.05.018 PMC9263322

[44] K. Reinhard , B. Rengstl , P. Oehm , K. Michel , A. Billmeier , N. Hayduk , O. Klein , K. Kuna , Y. Ouchan , S. Wöll , E. Christ , D. Weber , M. Suchan , T. Bukur , M. Birtel , V. Jahndel , K. Mroz , K. Hobohm , L. Kranz , M. Diken , K. Kühlcke , Ö. Türeci , U. Sahin , Science 2020, 367, 446. 31896660 10.1126/science.aay5967

[45] J. D. Beck , D. Reidenbach , N. Salomon , U. Sahin , Ö. Türeci , M. Vormehr , L. M. Kranz , Mol. Cancer 2021, 20, 69. 33858437 10.1186/s12943-021-01348-0 PMC8047518

[46] C. Ye , D. Brand , S. G. Zheng , Signal Transduction Targeted Ther 2018, 3, 2. 10.1038/s41392-017-0002-5 PMC5837126 29527328

[47] M. M. Melssen , N. D. Sheybani , K. M. Leick , C. L. Slingluff, Jr. , J. Immunother. Cancer 2023, 11, e006401. 37072352 10.1136/jitc-2022-006401 PMC10124321

[48] N. M. Anderson , M. C. Simon , Curr. Biol. 2020, 30, R921. 32810447 10.1016/j.cub.2020.06.081 PMC8194051

[49] J. Winkler , A. Abisoye‐Ogunniyan , K. J. Metcalf , Z. Werb , Nat. Commun. 2020, 11, 5120. 33037194 10.1038/s41467-020-18794-x PMC7547708

[50] S. Maman , I. P. Witz , Nat. Rev. Cancer 2018, 18, 359. 29700396 10.1038/s41568-018-0006-7

[51] F. Wu , J. Yang , J. Liu , Ye Wang , J. Mu , Q. Zeng , S. Deng , H. Zhou , Signal Transduction Targeted Ther 2021, 6, 218. 10.1038/s41392-021-00641-0 PMC8190181 34108441

[52] E. Sahai , I. Astsaturov , E. Cukierman , D. G. Denardo , M. Egeblad , R. M. Evans , D. Fearon , F. R. Greten , S. R. Hingorani , T. Hunter , R. O. Hynes , R. K. Jain , T. Janowitz , C. Jorgensen , A. C. Kimmelman , M. G. Kolonin , R. G. Maki , R. S. Powers , E. Puré , D. C. Ramirez , R. Scherz‐Shouval , M. H. Sherman , S. Stewart , T. D. Tlsty , D. A. Tuveson , F. M. Watt , V. Weaver , A. T. Weeraratna , Z. Werb , Nat. Rev. Cancer 2020, 20, 174. 31980749 10.1038/s41568-019-0238-1 PMC7046529

[53] K. Kessenbrock , V. Plaks , Z. Werb , Cell 2010, 141, 52. 20371345 10.1016/j.cell.2010.03.015 PMC2862057

[54] a) S. Fernandes , J. O.‐D. L. Cruz , S. Morazzo , F. Niro , M. Cassani , H. Duríková , A. Caravella , P. Fiore , G. Azzato , G. De Marco , A. Lauria , V. Izzi , V. Bosáková , J. Fric , P. Filipensky , G. Forte , Matrix Biol. 2023, 10.1016/j.matbio.2023.11.001; 37944712

[55] a) E. Henke , R. Nandigama , S. Ergün , Front. Mol. Biosci. 2019, 6, 160; 32118030 10.3389/fmolb.2019.00160 PMC7025524

[56] H. Salmon , E. Donnadieu , Oncoimmunology 2012, 1, 992. 23162783 10.4161/onci.20239 PMC3489771

[57] S. K. Kim , S. W. Cho , Front. Pharmacol. 2022, 13, 868695. 35685630 10.3389/fphar.2022.868695 PMC9171538

[58] G. Bergers , L. E. Benjamin , Nat. Rev. Cancer 2003, 3, 401. 12778130 10.1038/nrc1093

[59] Y. Zhao , J. Li , K. K. Ting , J. Chen , P. Coleman , K. Liu , L. Wan , T. Moller , M. A. Vadas , J. R. Gamble , Cancer Lett 2021, 496, 1. 32991950 10.1016/j.canlet.2020.09.026

[60] M. Binnewies , E. W. Roberts , K. Kersten , V. Chan , D. F. Fearon , M. Merad , L. M. Coussens , D. I. Gabrilovich , S. Ostrand‐Rosenberg , C. C. Hedrick , R. H. Vonderheide , M. J. Pittet , R. K. Jain , W. Zou , T. K. Howcroft , E. C. Woodhouse , R. A. Weinberg , M. F. Krummel , Nat. Med. 2018, 24, 541. 29686425 10.1038/s41591-018-0014-x PMC5998822

[61] S.‐B. Yong , S. Ramishetti , M. Goldsmith , Y. Diesendruck , I. Hazan‐Halevy , S. Chatterjee , G. Somu Naidu , A. Ezra , D. Peer , Adv. Mater. 2022, 34, 2106350. 10.1002/adma.202106350 35044699

[62] P. Zhang , A. Rashidi , J. Zhao , C. Silvers , H. Wang , B. Castro , A. Ellingwood , Yu Han , A. Lopez‐Rosas , M. Zannikou , C. Dmello , R. Levine , T. Xiao , A. Cordero , A. M. Sonabend , I. V. Balyasnikova , C. Lee‐Chang , J. Miska , M. S. Lesniak , Nat. Commun. 2023, 14, 1610. 36959214 10.1038/s41467-023-37328-9 PMC10036562

[63] I. Melero , A. Rouzaut , G. T. Motz , G. Coukos , Cancer Discovery 2014, 4, 522. 24795012 10.1158/2159-8290.CD-13-0985 PMC4142435

[64] J. Galon , D. Bruni , Nat. Rev. Drug Discovery 2019, 18, 197. 30610226 10.1038/s41573-018-0007-y

[65] a) W. H. Fridman , F. Pagès , C. Sautès‐Fridman , J. Galon , Nat. Rev. Cancer 2012, 12, 298; 22419253 10.1038/nrc3245

[66] M. M. Marit , D. S. Natasha , M. L. Katie , C. L. Slingluff, Jr. , J. Immunother. Cancer 2023, 11, e006401.37072352

[67] B. Weigelin , A. T. den Boer , E. Wagena , K. Broen , H. Dolstra , R. J. de Boer , C. G. Figdor , J. Textor , P. Friedl , Nat. Commun 2021, 12, 5217. 34471116 10.1038/s41467-021-25282-3 PMC8410835

[68] a) H. C. Pruitt , D. Lewis , M. Ciccaglione , S. Connor , Q. Smith , J. W. Hickey , J. P. Schneck , S. Gerecht , Matrix Biol. 2020, 85–86, 147; 10.1016/j.matbio.2019.02.003 PMC6697628 30776427

[69] H. Salmon , K. Franciszkiewicz , D. Damotte , M.‐C. Dieu‐Nosjean , P. Validire , A. Trautmann , F. Mami‐Chouaib , E. Donnadieu , J. Clin. Invest. 2012, 122, 899. 22293174 10.1172/JCI45817 PMC3287213

[70] M. Conroy , J. Naidoo , Nat. Commun. 2022, 13, 392. 35046403 10.1038/s41467-022-27960-2 PMC8770784

[71] Y.‐D. Yan , Y. Zhao , C. Zhang , J. Fu , Y.‐J. Su , X.‐L. Cui , E.‐L. Ma , B.‐L. Liu , Z.‐C. Gu , H.‐W. Lin , eClinicalMedicine 2022, 50, 101535. 35812997 10.1016/j.eclinm.2022.101535 PMC9256649

[72] S. Champiat , L. Tselikas , S. Farhane , T. Raoult , M. Texier , E. Lanoy , C. Massard , C. Robert , S. Ammari , T. De Baère , A. Marabelle , Clin. Cancer Res. 2021, 27, 665. 32943460 10.1158/1078-0432.CCR-20-0473

[73] S. Zhou , W. Chen , J. Cole , G. Zhu , Med. Drug Discovery 2020, 6, 100023. 10.1016/j.medidd.2020.100023 PMC8320672 34337382

[74] A. Ray , M. A. Williams , S. M. Meek , R. C. Bowen , K. F. Grossmann , R. H. I. Andtbacka , T. L. Bowles , J. R. Hyngstrom , S. A. Leachman , D. Grossman , G. M. Bowen , S. L. Holmen , M. W. Vanbrocklin , G. Suneja , H. T. Khong , Oncotarget 2016, 7, 64390. 27391442 10.18632/oncotarget.10453 PMC5325451

[75] S. Y. Van Der Zanden , J. J. Luimstra , J. Neefjes , J. Borst , H. Ovaa , Trends Immunol 2020, 41, 493. 32381382 10.1016/j.it.2020.04.004

[76] R. Fröbom , E. Berglund , D. Berglund , I.‐L. Nilsson , J. Åhlén , K. Von Sivers , C. Linder‐Stragliotto , P. Suenaert , A. Karlsson‐Parra , R. Bränström , Cancer Immunol. Immunother. 2020, 69, 2393. 32535637 10.1007/s00262-020-02625-5 PMC7568699

[77] J.‐Q. Liu , C. Zhang , X. Zhang , J. Yan , C. Zeng , F. Talebian , K. Lynch , W. Zhao , X. Hou , S. Du , D. D. Kang , B. Deng , D. W. Mccomb , X.‐F. Bai , Y. Dong , J. Controlled Release 2022, 345, 306. 10.1016/j.jconrel.2022.03.021 PMC9133152 35301053

[78] S. L. Hewitt , D. Bailey , J. Zielinski , A. Apte , F. Musenge , R. Karp , S. Burke , F. Garcon , A. Mishra , S. Gurumurthy , A. Watkins , K. Arnold , J. Moynihan , E. Clancy‐Thompson , K. Mulgrew , G. Adjei , K. Deschler , D. Potz , G. Moody , D. A. Leinster , S. Novick , M. Sulikowski , C. Bagnall , P. Martin , J.‐M. Lapointe , H. Si , C. Morehouse , M. Sedic , R. W. Wilkinson , R. Herbst , et al., Clin. Cancer Res. 2020, 26, 6284. 32817076 10.1158/1078-0432.CCR-20-0472

[79] Y. Li , Z. Su , W. Zhao , X. Zhang , N. Momin , C. Zhang , K. D. Wittrup , Y. Dong , D. J. Irvine , R. Weiss , Nat. Cancer 2020, 1, 882. 34447945 10.1038/s43018-020-0095-6 PMC8386348

[80] S. Ndeupen , Z. Qin , S. Jacobsen , A. Bouteau , H. Estanbouli , B. Z. Igyártó , iScience 2021, 24, 103479. 34841223 10.1016/j.isci.2021.103479 PMC8604799

[81] R. Kedmi , N. Ben‐Arie , D. Peer , Biomaterials 2010, 31, 6867. 20541799 10.1016/j.biomaterials.2010.05.027

[82] a) L. A. Jackson , E. J. Anderson , N. G. Rouphael , P. C. Roberts , M. Makhene , R. N. Coler , M. P. Mccullough , J. D. Chappell , M. R. Denison , L. J. Stevens , A. J. Pruijssers , A. Mcdermott , B. Flach , N. A. Doria‐Rose , K. S. Corbett , K. M. Morabito , S. O'dell , S. D. Schmidt , P. A. Swanson , M. Padilla , J. R. Mascola , K. M. Neuzil , H. Bennett , W. Sun , E. Peters , M. Makowski , J. Albert , K. Cross , W. Buchanan , R. Pikaart‐Tautges , et al., N. Engl. J. Med. 2020, 383, 1920; 32663912 10.1056/NEJMoa2022483 PMC7377258

[83] M. Estapé Senti , C. A. De Jongh , K. Dijkxhoorn , J. J. F. Verhoef , J. Szebeni , G. Storm , C. E. Hack , R. M. Schiffelers , M. H. Fens , P. Boross , J. Controlled Release 2022, 341, 475. 10.1016/j.jconrel.2021.11.042 34890719

[84] J. Shi , P. W. Kantoff , R. Wooster , O. C. Farokhzad , Nat. Rev. Cancer 2017, 17, 20. 27834398 10.1038/nrc.2016.108 PMC5575742

[85] J. Song , Y. Ju , T. H. Amarasena , Z. Lin , S. Mettu , J. Zhou , Md. A Rahim , C.‐S. Ang , C. Cortez‐Jugo , S. J. Kent , F. Caruso , ACS Nano 2021, 15, 10025. 34009935 10.1021/acsnano.1c01642

[86] Y. Ju , H. G. Kelly , L. F. Dagley , A. Reynaldi , T. E. Schlub , S. K. Spall , C. A. Bell , J. Cui , A. J. Mitchell , Z. Lin , A. K. Wheatley , K. J. Thurecht , M. P. Davenport , A. I. Webb , F. Caruso , S. J. Kent , ACS Nano 2020, 14, 15723. 33112593 10.1021/acsnano.0c06679

[87] a) S. S. Nogueira , A. Schlegel , K. Maxeiner , B. Weber , M. Barz , M. A. Schroer , C. E. Blanchet , D. I. Svergun , S. Ramishetti , D. Peer , P. Langguth , U. Sahin , H. Haas , ACS Appl. Nano Mater 2020, 3, 10634;

[88] C.‐P. Day , G. Merlino , T. Van Dyke , Cell 2015, 163, 39. 26406370 10.1016/j.cell.2015.08.068 PMC4583714

[89] K. J. Hassett , J. Higgins , A. Woods , B. Levy , Y. Xia , C. J. Hsiao , E. Acosta , Ö. Almarsson , M. J. Moore , L. A. Brito , J. Controlled Release 2021, 335, 237. 10.1016/j.jconrel.2021.05.021 34019945

[90] K. Lam , P. Schreiner , A. Leung , P. Stainton , S. Reid , E. Yaworski , P. Lutwyche , J. Heyes , Adv. Mater. 2023, 35, 2211420. 10.1002/adma.202211420 36972555

[91] J. Chuprin , H. Buettner , M. O. Seedhom , D. L. Greiner , J. G. Keck , F. Ishikawa , L. D. Shultz , M. A. Brehm , Nat. Rev. Clin. Oncol. 2023, 20, 192. 36635480 10.1038/s41571-022-00721-2 PMC10593256

[92] T. M. Allen , M. A. Brehm , S. Bridges , S. Ferguson , P. Kumar , O. Mirochnitchenko , K. Palucka , R. Pelanda , B. Sanders‐Beer , L. D. Shultz , L. Su , M. Prabhudas , Nat. Immunol. 2019, 20, 770. 31160798 10.1038/s41590-019-0416-z PMC7265413

[93] M. Z. C. Hatit , M. P. Lokugamage , C. N. Dobrowolski , K. Paunovska , H. Ni , K. Zhao , D. Vanover , J. Beyersdorf , H. E. Peck , D. Loughrey , M. Sato , A. Cristian , P. J. Santangelo , J. E. Dahlman , Nat. Nanotechnol. 2022, 17, 310. 35132167 10.1038/s41565-021-01030-y PMC9082280

[94] K.‐T. Jin , W.‐L. Du , H.‐R. Lan , Y.‐Y. Liu , C.‐S. Mao , J.‐L. Du , X.‐Z. Mou , Cancer Sci. 2021, 112, 2592. 33938090 10.1111/cas.14934 PMC8253285

[95] M. B. Carper , S. Troutman , B. L. Wagner , K. M. Byrd , S. R. Selitsky , K. Parag‐Sharma , E. C. Henry , W. Li , J. S. Parker , S. A. Montgomery , J. L. Cleveland , S. E. Williams , J. L. Kissil , D. N. Hayes , A. L. Amelio , Cell Rep 2019, 29, 1660. 31693903 10.1016/j.celrep.2019.10.005 PMC6870917

[96] a) N. K. Verma , B. H. S. Wong , Z. S. Poh , A. Udayakumar , R. Verma , R. K. J. Goh , S. P. Duggan , V. G. Shelat , K. G. Chandy , N. F. Grigoropoulos , EBioMedicine 2022, 83, 104216; 35986950 10.1016/j.ebiom.2022.104216 PMC9403334

[97] G. Raghu , K. K. Brown , H. R. Collard , V. Cottin , K. F. Gibson , R. J. Kaner , D. J. Lederer , F. J. Martinez , P. W. Noble , J. W. Song , A. U. Wells , T. P. M. Whelan , W. Wuyts , E. Moreau , S. D. Patterson , V. Smith , S. Bayly , J. W. Chien , Qi Gong , J. J. Zhang , T. G. O'riordan , Lancet Respir. Med. 2017, 5, 22. 27939076 10.1016/S2213-2600(16)30421-0

[98] N. Ikenaga , Z.‐W. Peng , K. A. Vaid , S. B. Liu , S. Yoshida , D. Y. Sverdlov , A. Mikels‐Vigdal , V. Smith , D. Schuppan , Y. V. Popov , Gut 2017, 66, 1697. 28073888 10.1136/gutjnl-2016-312473 PMC5561383

[99] T. E. Sutherland , D. P. Dyer , J. E. Allen , Science 2023, 379, eabp8964. 36795835 10.1126/science.abp8964

[100] T. P. Rygiel , E. H. Stolte , T. De Ruiter , M. L. Van De Weijer , L. Meyaard , Mol. Immunol. 2011, 49, 402. 21955987 10.1016/j.molimm.2011.09.006

[101] D. H. Peng , B. L. Rodriguez , L. Diao , L. Chen , J. Wang , L. A. Byers , Y. Wei , H. A. Chapman , M. Yamauchi , C. Behrens , G. Raso , L. M. S. Soto , E. R. P. Cuentes , I. I. Wistuba , J. M. Kurie , D. L. Gibbons , Nat. Commun. 2020, 11, 4520. 32908154 10.1038/s41467-020-18298-8 PMC7481212

[102] A. P. Deshmukh , S. V. Vasaikar , K. Tomczak , S. Tripathi , P. Den Hollander , E. Arslan , P. Chakraborty , R. Soundararajan , M. K. Jolly , K. Rai , H. Levine , S. A. Mani , Proc. Natl. Acad. Sci. USA 2021, 118, e2102050118. 33941680 10.1073/pnas.2102050118 PMC8126782

[103] a) T. R. Cox , Nat. Rev. Cancer 2021, 21, 217; 33589810 10.1038/s41568-020-00329-7

[104] a) G. Nardone , J. Oliver‐De La Cruz , J. Vrbsky , C. Martini , J. Pribyl , P. Skládal , M. Pesl , G. Caluori , S. Pagliari , F. Martino , Z. Maceckova , M. Hajduch , A. Sanz‐Garcia , N. M. Pugno , G. B. Stokin , G. Forte , Nat. Commun. 2017, 8, 15321; 28504269 10.1038/ncomms15321 PMC5440673

[105] Y.‐W. Shen , Y.‐D. Zhou , H.‐Z. Chen , X. Luan , W.‐D. Zhang , Trends Cancer 2021, 7, 511. 33358571 10.1016/j.trecan.2020.12.001

[106] a) F. Schütze , F. Röhrig , S. Vorlová , S. Gätzner , A. Kuhn , S. Ergün , E. Henke , Sci. Rep. 2015, 5, 17576; 26620400 10.1038/srep17576 PMC4665164

[107] a) G. Vlahovic , Z. N. Rabbani , J. E. Herndon , M. W. Dewhirst , Z. Vujaskovic , Br. J. Cancer 2006, 95, 1013; 17003785 10.1038/sj.bjc.6603366 PMC2360712

[108] Di Zhang , G. Wang , X. Yu , T. Wei , L. Farbiak , L. T. Johnson , A. M. Taylor , J. Xu , Yi Hong , H. Zhu , D. J. Siegwart , Nat. Nanotechnol. 2022, 17, 777. 35551240 10.1038/s41565-022-01122-3 PMC9931497

[109] a) D. J. McGrail , Q. M. Kieu , M. R. Dawson , J. Cell Sci. 2014, 127, 2621; 24741068 10.1242/jcs.144378 PMC4058108

[110] J. Drost , H. Clevers , Nat. Rev. Cancer 2018, 18, 407. 29692415 10.1038/s41568-018-0007-6

[111] a) E. Driehuis , K. Kretzschmar , H. Clevers , Nat. Protoc. 2020, 15, 3380; 32929210 10.1038/s41596-020-0379-4

[112] a) K. T. Lawlor , J. M. Vanslambrouck , J. W. Higgins , A. Chambon , K. Bishard , D. Arndt , P. X. Er , S. B. Wilson , S. E. Howden , K. S. Tan , F. Li , L. J. Hale , B. Shepherd , S. Pentoney , S. C. Presnell , A. E. Chen , M. H. Little , Nat. Mat. 2021, 20, 260; 10.1038/s41563-020-00853-9 PMC7855371 33230326

[113] A. Mullard , Nat. Rev. Drug Discovery 2023, 22, 175. 36797431 10.1038/d41573-023-00030-y

[114] J. T. Neal , X. Li , J. Zhu , V. Giangarra , C. L. Grzeskowiak , J. Ju , I. H. Liu , S. H. Chiou , A. A. Salahudeen , A. R. Smith , B. C. Deutsch , L. Liao , A. J. Zemek , F. Zhao , K. Karlsson , L. M. Schultz , T. J. Metzner , L. D. Nadauld , Y. Y. Tseng , S. Alkhairy , C. Oh , P. Keskula , D. Mendoza‐Villanueva , F. M. De La Vega , P. L. Kunz , J. C. Liao , J. T. Leppert , J. B. Sunwoo , C. Sabatti , J. S. Boehm , et al., Cell 1972, 2018, 175, 10.1016/j.cell.2018.11.021 PMC6656687 30550791

[115] S. Tsai , L. Mcolash , K. Palen , B. Johnson , C. Duris , Q. Yang , M. B. Dwinell , B. Hunt , D. B. Evans , J. Gershan , M. A. James , BMC Cancer 2018, 18, 335. 29587663 10.1186/s12885-018-4238-4 PMC5870823

[116] J. F. Dekkers , M. Alieva , A. Cleven , F. Keramati , A. K. L. Wezenaar , E. J. Van Vliet , J. Puschhof , P. Brazda , I. Johanna , A. D. Meringa , H. G. Rebel , M.‐B. Buchholz , M. Barrera Román , A. L. Zeeman , S. De Blank , D. Fasci , M. H. Geurts , A. M. Cornel , E. Driehuis , R. Millen , T. Straetemans , M. J. T. Nicolasen , T. Aarts‐Riemens , H. C. R. Ariese , H. R. Johnson , R. L. Van Ineveld , F. Karaiskaki , O. Kopper , Y. E. Bar‐Ephraim , K. Kretzschmar , et al., Nat. Biotechnol. 2023, 41, 60. 35879361 10.1038/s41587-022-01397-w PMC9849137

[117] J. J. Han , Artif. Organs 2023, 47, 449. 36762462 10.1111/aor.14503

[118] I. Yakavets , A. Francois , L. Lamy , M. Piffoux , F. Gazeau , C. Wilhelm , V. Zorin , A. K. A. Silva , L. Bezdetnaya , J. Nanobiotechnol. 2021, 19, 3. 10.1186/s12951-020-00743-x PMC7789590 33407564

[119] M. Niora , D. Pedersbæk , R. Münter , M F De V Weywadt , Y. Farhangibarooji , T. L. Andresen , J. B. Simonsen , L. Jauffred , ACS Omega 2020, 5, 21162. 32875252 10.1021/acsomega.0c02879 PMC7450641

[120] J. Chen , Z. Ye , C. Huang , M. Qiu , D. Song , Y. Li , Q. Xu , Proc. Natl. Acad. Sci. U. S. A. 2022, 119, e2207841119. 35969778 10.1073/pnas.2207841119 PMC9407666

